# Putative Inhibitors of SARS-CoV-2 Main Protease from A Library of Marine Natural Products: A Virtual Screening and Molecular Modeling Study

**DOI:** 10.3390/md18040225

**Published:** 2020-04-23

**Authors:** Davide Gentile, Vincenzo Patamia, Angela Scala, Maria Teresa Sciortino, Anna Piperno, Antonio Rescifina

**Affiliations:** 1 Department of Drug Sciences, University of Catania, V.le A. Doria, 95125 Catania, Italy; vincenzo.patamia@unict.it; 2 Department of Chemical, Biological, Pharmaceutical, and Environmental Sciences, University of Messina, V.le F. Stagno d’Alcontres 31, 98166 Messina, Italy; ascala@unime.it (A.S.); mtsciortino@unime.it (M.T.S.); apiperno@unime.it (A.P.); 3 Consorzio Interuniversitario Nazionale di ricerca in Metodologie e Processi Innovativi di Sintesi (CINMPS), Via E. Orabona, 4, 70125 Bari, Italy

**Keywords:** COVID-19, SARS-CoV-2, marine natural product, virtual screening, docking

## Abstract

The current emergency due to the worldwide spread of the COVID-19 caused by the new severe acute respiratory syndrome coronavirus 2 (SARS-CoV-2) is a great concern for global public health. Already in the past, the outbreak of severe acute respiratory syndrome (SARS) in 2003 and Middle Eastern respiratory syndrome (MERS) in 2012 demonstrates the potential of coronaviruses to cross-species borders and further underlines the importance of identifying new-targeted drugs. An ideal antiviral agent should target essential proteins involved in the lifecycle of SARS-CoV. Currently, some HIV protease inhibitors (i.e., Lopinavir) are proposed for the treatment of COVID-19, although their effectiveness has not yet been assessed. The main protease (M^pro^) provides a highly validated pharmacological target for the discovery and design of inhibitors. We identified potent M^pro^ inhibitors employing computational techniques that entail the screening of a Marine Natural Product (MNP) library. MNP library was screened by a hyphenated pharmacophore model, and molecular docking approaches. Molecular dynamics and re-docking further confirmed the results obtained by structure-based techniques and allowed this study to highlight some crucial aspects. Seventeen potential SARS-CoV-2 M^pro^ inhibitors have been identified among the natural substances of marine origin. As these compounds were extensively validated by a consensus approach and by molecular dynamics, the likelihood that at least one of these compounds could be bioactive is excellent.

## 1. Introduction

The new coronavirus, designated as severe acute respiratory syndrome coronavirus 2 (SARS-CoV-2), was first identified in Wuhan, China, in December 2019 [1]. SARS-CoV-2 belongs to the family of Coronaviridae, single-stranded RNA virus (+ssRNA) that spreads widely among humans and other mammals, causing a wide range of infections from common cold symptoms to fatal diseases, such as severe respiratory syndrome [2,3]. The worldwide mortality rate of this new virus appears to be around 3.4% (https://www.worldometers.info/coronavirus/coronavirus-death-rate/#who-03-03-20), which is much lower than the 9.6% mortality rate of SARS-CoV-1 (https://www.worldometers.info/coronavirus/coronavirus-death-rate/#comparison). It should be considered that the majority of fatal cases are vulnerable populations with comorbidities such as immunosuppression, diabetes, or heart disease. The primary issue all over the world is the high human-to-human transmission that has led to the spread of its outbreaks in many countries [4]. 

The World Health Organization (WHO) classified the COVID-19 outbreak as a pandemic on 11 March 2020. To date, SARS-CoV-2 has infected more than 1.6 million people all around the world, while more than 96,000 have been killed (data from https://www.worldometers.info/coronavirus/, accessed on 10 April 2020), indicating that the outbreak is a serious public health concern [1].

Unfortunately, the number of infected continues to grow, and no drugs have been approved to be effective. Therefore, the need to discover and develop drugs for the treatment of the Coronavirus Disease 2019 (COVID-19) is urgent. Remdesivir, an antiviral agent designed for the Ebola virus disease, was suggested to be a potential SARS-CoV-2 RNA-dependent RNA polymerase (RdRp) inhibitor by Liu et al., who also suggested three other possible inhibitors of SARS-CoV-2 target enzymes [5]. Many research teams have exploited the major protease (M^pro^) of SARS-CoV-2, also named chymotrypsin-like protease (3CL^pr^) [6], as a potential drug target to fight COVID-19. Sequence alignment revealed that the SARS-CoV-2 M^pro^ shares a 96% similarity to that of SARS-CoV-1 [7]. For example, Jiang and colleagues identified 30 drugs and compounds as SARS-CoV-2 M^pro^ inhibitors through protein modeling and virtual screening [8], which represents rapid progress in the way of dealing with the crisis. The SARS-CoV-2 M^pro^ and SARS-CoV-1 M^pro^ structures are quite similar, the main difference being the surface of the proteins, where 12 different amino acids are located. Both enzymes consist of three domains; the domains I (residues 1–101) and II (residues 102–184) consist of an antiparallel *β*-barrel, and, for enzymatic activity, the α-helical domain III is required (residues 201–301) [9,10,11].

Furthermore, both SARS-CoV-2 M^pro^ and SARS-CoV-1 M^pro^ have a structure similar to that of the cysteine proteases, although the third catalytic residue is missing at their active site. Their active site includes a catalytic dyad, His41 and Cys145, and a particularly stable water molecule that forms at least three hydrogen-bonding interactions with the surrounding residues, including a catalytic histidine, which corresponds to the position of the third catalytic element [10,11]. It should also be noted that one of the different amino acids in SARS-CoV-2 M^pro^, namely Ser46, is located on the Cys44–Pro52 loop that flanks the cavity of the active site. Another fundamental difference between SARS-CoV-2 M^pro^ and SARS-CoV-1 M^pro^ is given by the different sizes and shapes of the external pockets found in the two systems. Surprisingly, the volume of the external pockets of the SARS-CoV-2 M^pro^ is, on average, half that of the SARS-CoV-1 M^pro^. Thus, considering the similarity of the two structures, it can be assumed that there may be large differences between the accessibility to the binding cavity and/or the accommodation of the shape of the cavity in response to an inhibitor that can be bound. Moreover, the SARS-CoV-1 M^pro^ apo structure has shown the largest outer pocket respect to both ligand-bounded M^pro^ structures, suggesting that the SARS M^pro^ binding cavity is highly flexible and shows significant changes in both volume and shape after ligand binding [10,11].

These features can be exploited in the design of lead compounds with a potentially broad spectrum of activity. Generally, a suitable substrate can be converted to a good inhibitor by replacement of a part of the substrate sequence that binds directly to the active site of the protease, reversibly or irreversibly, with the chemical warhead targeting the catalytic mechanism. Peptide inhibitors were designed by attaching a chemical warhead (i.e., Michael acceptors, aldehydes, ketones, etc.) to a peptide that mimics the natural substrate [12]. These inhibitors act through a two-step procedure, wherein they first bind and form a non-covalent complex with the enzyme such that the warhead is preorganized near the catalytic residue. Subsequently, the nucleophilic attack occurs at the cysteine, leading to the formation of the covalent bond. Some peptidomimetic derivatives contain Michael acceptors as warheads and are an important class of cysteine protease inhibitors. The cysteine residue undergoes 1,4-addition to the inhibitor at the Michael acceptor warhead group, and the subsequent protonation of the α-carbanion results in the irreversible inhibition of the enzyme [12]. Our work aims to perform a virtual screening against the SARS-CoV-2 M^pro^ binding site using the library of Marine Natural Products (MNP). Many MNP have been detected as having various biological activities; peptides isolated from fish as well as algal polysaccharides have been reported to have anti-cancer, anticoagulant, and inhibitor activities. Marine bacteria and fish oils contain a considerable amount of omega-3 fatty acids, whereas seaweeds and shellfish, such as crustaceans, have potent antioxidants, including carotenoids and phenolic compounds [13]. On a pharmacophore model, built by Pharmit server (http://pharmitcsb.pitt.edu/) [14] starting from the SARS-CoV-2 M^pro^ (PDB ID: 6LU7) and with the complexed ligand N3 (PRD_002214) structure employed as input, the virtual screening on the 164,952 conformers of the 14,064 molecules contained in the MNP library was carried out. Among these, 180 molecules were docked using AutoDock Vina software. Finally, we conducted a parallel docking study with AutoDock4 and molecular dynamics (MD) simulation studies, of 10 ns each, on the 17 molecules that showed the most promising results in terms of inhibitory activity.

## 2. Results and Discussion

### 2.1. Catalytic Site of the SARS-CoV-2 M^pro^

**Figure 1:**
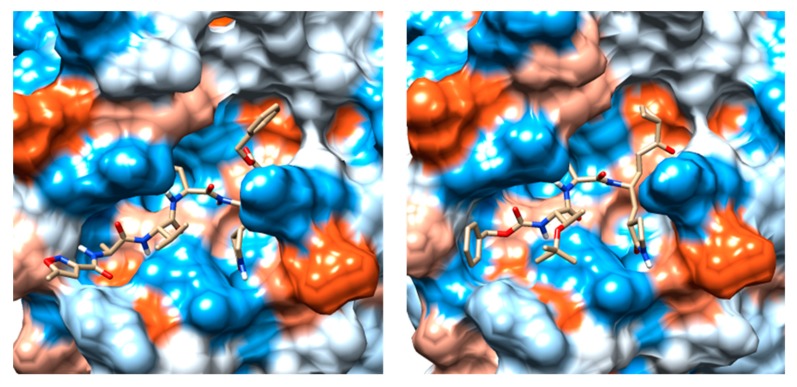
(**a**) Comparison between the binding pockets of the crystalline structure of the N3 inhibitor linked to the severe acute respiratory syndrome coronavirus 2 (SARS-CoV-2) main protease (M^pro^) (PDB ID: 6LU7); (**b**) the crystal structure of TG-0205486 inhibitor bound to the severe acute respiratory syndrome coronavirus (SARS-CoV-1) M^pro^ (PDB ID: 2ZU5).

The crystal structure of SARS-CoV-2 M^pro^ (PDB ID 6LU7) in complex with the N3 (ID PRD_002214) inhibitor was previously solved by X-ray crystallography at a high resolution of 2.16 Å [8]. In this structure, the N3 peptide inhibitor is covalently bound with the Cys145 residue (Figure 1). Moreover, the surface topology of the active site inherent both SARS-CoV-2 M^pro^ and SARS-CoV-1 M^pro^ proteins shows some differences, consisting in more sub-cavities and a smaller volume (337.2 A^3^ vs. 447.7 A^3^) for the first one (Figure 1). It should be noted that the volume of the crystallized ligand influences the volume of the catalytic pocket. Moreover, the high flexibility of the catalytic site plays an essential role in the volume of the M^pro^ pocket. Consequently, inhibitors of the SARS-CoV-1 M^pro^ might present altered binding affinities for the SARS-CoV-2 M^pro^.

### 2.2. Pharmacophore Model

A pharmacophore defines the essential features of interactions, including the spatial arrangement of each interaction in the bond between the drug and the target [14]. Structure-based pharmacophore modeling usually uses the 3D-structure of a protein while it is interacting with its docked ligand. Therefore, pharmacophore is capable of incorporating more detailed information about regions available to the ligand to bind efficiently to its receptor.

**Figure 2:**
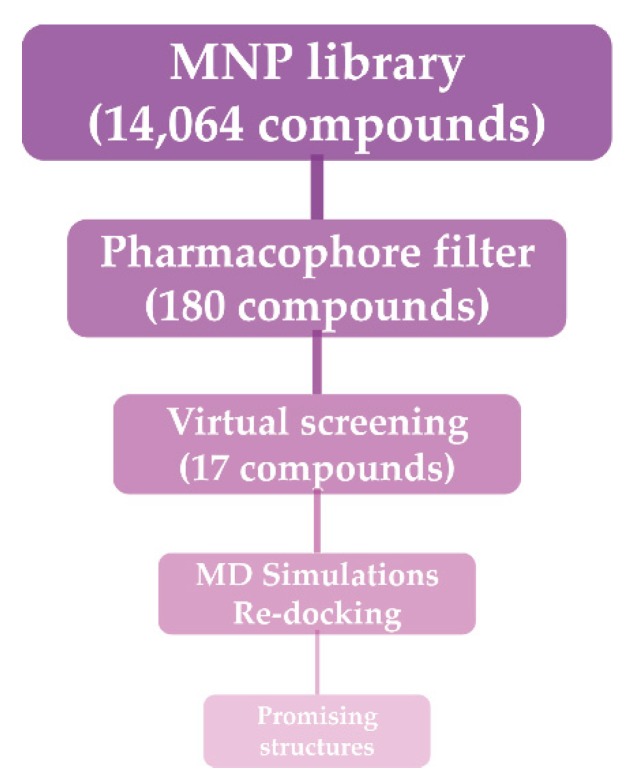
Workflow of the adopted molecular modeling procedure.

In drug design, the arrangement of steric and electronic characteristics of the pharmacophore is necessary to guarantee the finest molecular interactions with the respective biological target and to inhibit its biological function [15]. The use of multiple techniques, therefore, improves the probability of identifying a diverse set of lead compounds. To design a novel SARS-CoV-2 M^pro^ inhibitor, we sought new scaffolds from MNP using a pharmacophore model of the N3/SARS-CoV-2 M^pro^ co-crystallized structure. The workflow of the virtual screening strategy for the discovery of new scaffolds binding the catalytic site of SARS-CoV-2 M^pro^ is depicted in Figure 2. The 3D pharmacophore search was performed using the Pharmit server [14], which provides both pharmacophore and molecular shape search modalities as well as a ranking of results by energy minimization.

**Figure 3:**
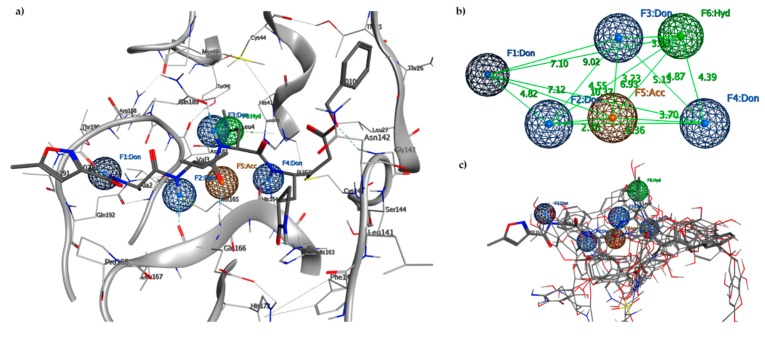
(**a**) Pharmacophore model generated by the Pharmit server, including hydrogen bond donors (DON) (blue spheres), negatively charged oxygen atom to represent a hydrogen bond acceptor (ACC) (orange sphere) and the hydrophobic center (HYD) (green sphere); (**b**) 3D spatial distribution of the six pharmacophore features; (**c**) Six features of the pharmacophore model on the 17 aligned compounds.

The proposed pharmacophore model is a binding-site-derived pharmacophore model, which includes the following pharmacophore features of ligands binding to the enzyme active site: three amide nitrogen atoms to represent hydrogen bond donors (DON), two negatively charged oxygen atoms (as in a carboxyl group) to represent a hydrogen bond acceptor (ACC), and the isopropyl group to represent a hydrophobic center (HYD) (Figure 3).

To decrease the pharmacophore selectivity for peptide ligands and also to favor non-peptide structures, the rays of every single point (from 0.5 to 0.8) were increased, while the other characteristics remained unchanged. The essential characteristics of the generated pharmacophore model included four hydrogen bond donors features, F1–F4, allocated in an opportune position to interact with residues Thr190, Glu166, Gnl189, and His164, respectively; one hydrogen bond acceptor feature, F5, that is positioned to interact with Glu166 residue; and one hydrophobic center feature, F6, that is related to the interactions of the isopropyl group of the N3 inhibitor located in the hydrophobic pocket. Points F2, F4, and F5 are aligned along with the peptide recognition site of the catalytic site with a distance of 6.30 Å between F2 and F4, while the hydrophobic pocket is located behind the triad at a distance of 6.93 Å with respect to F5 and 4.39 Å compared to F4. The hydrophobic cavity identified in F6 with a volume of 111.4 Å^3^ is capable of hosting phenyl residues as in the case of Pseudotheonamide D. The hydrophobic site in the HYD sub-pocket offered a good criterion for searching for ligands with hydrophobic residues with dimensions consistent with the pocket. An analysis of these ideal pharmacophore features enables the setting of threshold values for simple descriptors (bruisedness, distance to the cavity center, interaction energy) to reduce the number of pharmacophore features without losing crucial information.

The generated pharmacophore model was used to filter a vast library of MNP (14,064 molecules, 164,952 conformers). From this library, 770 conformers meet the criteria of the pharmacophore filter, and after an ulterior filter that retained only one conformer for molecule with an RMSD lower than 2 Å with respect to the co-crystallized N3 ligand, 197 structures remained. Peptide structures without peptidomimetic regions or that are unable to act as Michael acceptors were manually discarded, as they do not meet the pharmacophore criteria of protease inhibitors. The residual 180 molecules have undergone the docking procedure.

### 2.3. Molecular Docking and MD Simulation

Molecular docking algorithms are often calibrated on a training set of experimental ligand-protein complexes, and the accuracy of these docking programs is often highly dependent on the used training set [15]. In this case, due to the lack of known ligands, it is essential to confirm that the docking software used for virtual screening can replicate the binding mode of a known experimental inhibitor for the enzymes studied. Although neither an effective antiviral drug nor a vaccine against COVID-19 is currently available, several reports have indicated that HIV-1 protease inhibitors, such as Lopinavir, have the potential for designing SARS-CoV-2 protease ligands [16]. In the attempt to have reference values (positive control), we decided to consider both the N3 co-crystallized ligand within the catalytic site of SARS-CoV-2 M^pro^ (PDB ID: 6LU7) and Lopinavir as comparative standards for the molecular docking and MD simulation experiments.

Replication of the experimental binding pose by molecular docking confirmed the suitability of the docking algorithm for virtual screening. Autodock Vina software was able to accurately predict the pose of N3, with an RMSD of only 0.254 Å with respect to that of the co-crystallized one.

The 180 MNP structures filtered and selected according to the pharmacophore descriptors were separately docked into the SARS-CoV-2 M^pro^ binding site. The flexible virtual screening was performed using Autodock Vina to find the most favorable binding interactions, and the calculated free binding energies were reported in Table 1 for the best 17 compounds and in Table S1 for all the others. To further validate the pharmacophore model descriptors, validate the poses and binding energies, and comprehensively investigate the interactions of the new ligands within the catalytic site of the protease, we conducted a parallel docking study, with Autodock4, and MD simulations on those compounds (**1**–**17**) that showed a better affinity (Table 1). For all compounds, a re-docking was performed, using Autodock Vina, after MD simulation, taking the averaged pose of the last 3 ns.

The most promising inhibitors of the SARS-CoV-2 M^pro^ (Table 1, **1**–**17**) are primarily represented by a class of molecules called phlorotannins, oligomers of phloroglucinol (1,3,5-trihydroxybenzene), isolated from *Sargassum spinuligerum* brown alga [17]. Although most of these phlorotannins were identified in *Sargassum spinuligerum*, other species of *Sargassum* may also contain a large number of phlorotannins, including phlorethols, fuhalols, and fucophlorethols. [18]. Algae from the *Sargassum* family are used extensively in traditional Chinese medicine [17].

**Figure 4:**
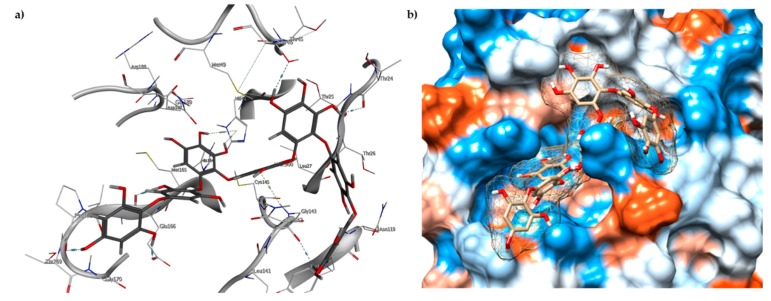
(**a**) Docked pose of **1** (grey, stick model) is shown with the binding pocket residues and interacting residues with M^pro^ (PDB ID: 6LU7); (**b**) View of **1** inside binding pocket in hydrophobic surface representation.

The results of the molecular docking showed that the tested compounds (**1**–**19**) had docking energies ranging from −14.6 to −10.7 kcal/mol (Table 1). Heptafuhalol A (**1**) showed the lowest docking energy (−14.60 kcal/mol). As shown in Figure 4, the hydroxyl groups in heptafuhalol A form an extensive network of H-bonds within the protease receptor site. The acceptor residues of hydrogen bonds are represented by Thr24, Ser46, Asn142, Glu166, and Pro168. Furthermore, *π*-hydrogen bonds with His41 and Gly143 residues, and hydrophobic interactions with Met49, Met65, Leu141, and Pro168, further stabilize the ligand-receptor complex. The results from the MD simulation of the heptafuhalol A/SARS-CoV-2 M^pro^ complex are shown in Figure S1. It is interesting to note that during the MD simulation the His41 residue, belonging to the catalytic dyad, establishes an H-bond with the hydroxyl residue of the ligand, with a distance of 1.97 Å and an average D–H–A angle of 177.8° after 10 ns of simulation, with a free energy of −18.0 kcal/mol from re-docking. The RMSDs of the overall structures (SARS-Cov-2 and compound **1**) with respect to the first ones at time 0 were analyzed and plotted during the 10 ns of MD simulation (Figure S1). The overall RMSD for the protein system appeared to have reached equilibrium after 800 ps, and the stabilization of the protein-ligand complex after 3 ns, keeping the broad network of hydrogen bonds of the complex constant. The ligand was linearly arranged with three aromatic systems along the back of the protein responsible for binding to the viral proteins. Total energy, RMSDs, ligand interactions, and docking binding poses of compounds **2**–**17**, N3, and Lopinavir were reported in Figures S2–S19.

We found the compounds **7**, **10**, and **11** to be the most active inhibitors, belonging to the family of phlorotannins, isolated in the brown algae *Ecklonia cava*. The latter is an edible seaweed, which has been recognized as a rich source of bioactive derivatives, mainly phlorotannins. These phlorotannins exhibit various beneficial biological activities such as antioxidant, anticancer, antidiabetic, anti-human immunodeficiency virus, antihypertensive, matrix metalloproteinase enzyme inhibition, hyaluronidase enzyme inhibition, radioprotective, and antiallergic [19,20,21,22,23,24,25]. Noteworthy, dieckol (**11**) has already reported as one of the most potent SARS-CoV-1 M^pro^ phlorotannin inhibitors (IC_50_ = 2.7 μM). Docking studies highlighted that interactions between dieckol and the amino acid residues in the active site of M^pro^ are mainly constituted by a H-bonds network with a calculated binding energy of −11.76 kcal/mol [26], that is comparable to the energy found by us with the SARS-CoV-2 M^pro^.

**Figure 5:**
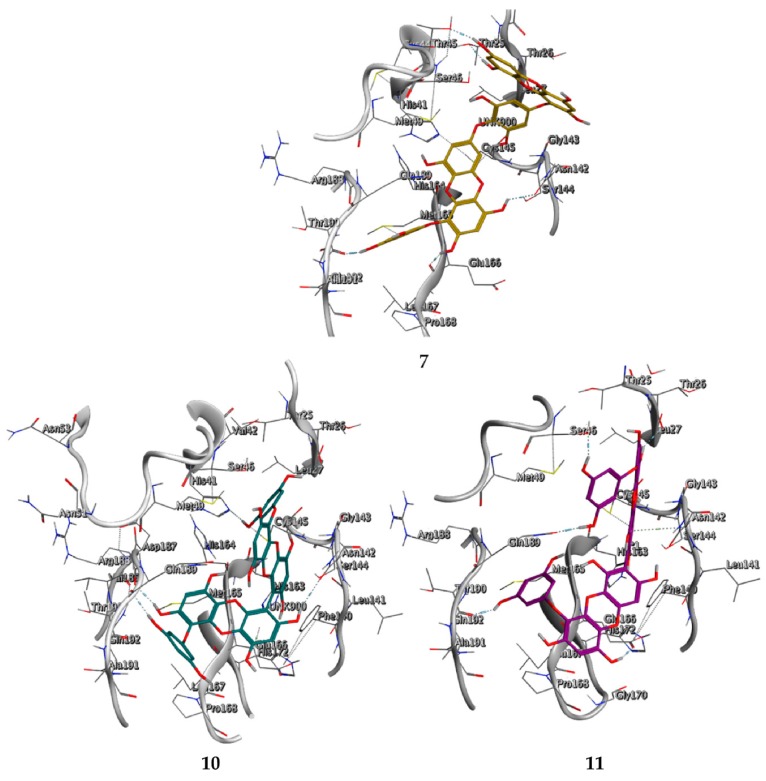
Interaction profile of the best-docked poses for compounds **7**, **10**, and **11**.

Although compound **7** has a better affinity (average binding energy = −12.9 kcal/mol), due to an extensive network of H-bonds, it is interesting to note that compounds **7** and **10** interact through H-bonds with the residues of the catalytic dyad His41 and Cys145 (Figure 5). In particular, 6,6’-bieckol forms an H-bond with the His41 residue with a D–A distance of 1.91 Å and a D–H–A angle of 176.0°. Dieckol shows a profile of hydrophobic interactions lower than the other two (**7** and **10**), showing interactions with the residues Leu27, Met41, Met49, Met165, Leu167, and Leu167.

**Figure 6:**
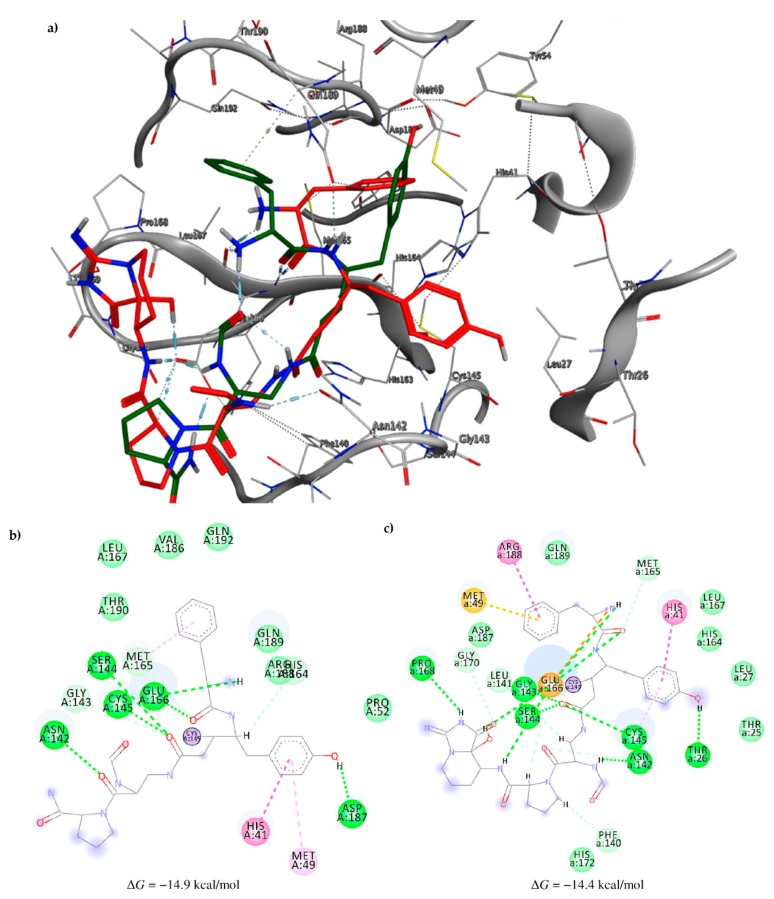
(**a**) Interaction profile of the best-docked poses for compounds **12** (green, stick model) and **17** (red, stick model). Binding site interactions between SARS-CoV-2 M^pro^ and pseudotheonamides (**b**) **12** and (**c**) **17**.

Among the compounds with the best-calculated activity against the SARS-CoV-2 M^pro^, also pseudotheonamide D (**12**) and pseudotheonamide C (**17**) emerged with a binding energy of −11.6 kcal/mol and −10.7 kcal/mol, respectively. In both cases, the formation of H-bonds with the residues Glu166 and Gln189 was fundamental for the recognition of viral peptides within the catalytic site. Furthermore, phenyl groups occupied the small hydrophobic pocket of the enzyme interacting with the residues Leu27, Met49, Phe140, and Leu167, so stabilizing the ligand-protein complex (Figure 6a).

It is interesting to note that these compounds could represent Michael acceptor covalent inhibitors. In fact, the conjugate bond is located at a distance of about 4 Å from Cys145, suggesting that both pseudotheonamides (**12** and **17**) could form a covalent bond with Cys145. These pseudotheonamides have been isolated from the marine sponge *Theonella swinhoei* and have shown good inhibitory activity on the serine protease [27]. Consequently, after having covalently linked the compounds **12** and **17** with the Cys145 residue, a short (2 ns) MD simulation was performed in order to stabilize the new complex. The lower energy system was further minimized, and covalent docking was performed. The binding energy of **12** and **17** is very similar (−14.9 kcal/mol and −14.4 kcal/mol, respectively) with a significant increase compared to the non-covalent interaction. The two compounds adopt a similar pose within the catalytic site, establishing H-bonds with the Asn142, Ser144, and Glu166 residues, while the benzyl groups settle into the hydrophobic pockets (Figure 6b,c). Peptidomimetic derivatives contain Michael acceptors as warheads are an essential class of cysteine protease inhibitors. In general, inhibitor design strategies involve the replacement of a substrate scissile amide bond with an appropriate Michael acceptor group. The cysteine residue undergoes 1,4-addition to the inhibitor at the Michael acceptor warhead group, and the subsequent protonation of the α-carbanion results in the irreversible inhibition of the enzyme [28,29,30].

Another class of promising M^pro^ inhibitors has been identified in flavonoids such as Apigenin-7-*O*-neohesperidoside, Luteolin-7-rutinoside, and Resinoside. These compounds are also widespread on terrestrial plants and in food waste with good anti-tumor, anti-inflammatory, and antioxidant activity [31,32,33,34,35]. Among these, Apigenin-7-*O*-neohesperidoside or Rhoifolin (whose structure belongs to flavone glycoside and its aglycone is apigenin, while the neohesperidose disaccharide constitutes the glycosidic structure) has the best binding energy (−12.39 kcal/mol). The docking pose of apigenin (Figure S8) shows H-bonds between the aromatic region and residues Leu141, Glu166, and Thr190, establishing a *π*-stacking interaction with Gln189. In SARS-CoV-1 M^pro^ it has been shown that the Gln189 mutation negatively affects inhibitory activity, suggesting that this area of the protein plays a key role in the binding interaction [36].

## 3. Materials and Methods

### 3.1. Dataset of Compounds

The chemical structures of the marine dataset were retrieved from Prof. Encinar website (http://docking.umh.es/downloaddb). The full list of the 180 molecules that passed the pharmacophore filter, including the MNP ID, contacting receptor residues, and Vina binding energy results, are available in Table 1 (compounds **1**–**17**, and in the supplementary material (Table S1).

### 3.2. Pharmacophore-based Virtual Screening and Database Preparation

The 3D pharmacophore search was performed using the Pharmit server (http://pharmit.csb.pitt.edu/) [14]. The pharmacophore model was constructed by Pharmit by inserting the SARS-CoV-2 enzyme (PDB 6LU7) and N3 ligand (PRD_002214) structures as input. Pharmit parameters for 3D-pharmacophore research have remained unchanged, except for the hydrophobic center (isopropyl group) with a radius of 1.5 A. This model was the basis for the virtual screening of the MNP library, which contained 14,064 molecules for a total of 164,952 conformers. The search was directed to select only one orientation for each conformation of the molecules. Compounds with an RMSD ≥ 2 Å than the N3 ligand were discarded. The remaining poses were minimized according to the functions of Pharmit.

### 3.3. Structures Preparation and Minimization

The structures of all the molecules used in this study were built using Marvin Sketch (18.24, ChemAxon Ltd., Budapest, Hungary). A first molecular mechanics energy minimization was used for 3D structures created from the SMLES; the Merck molecular force field (MMFF94) present in Marvin Sketch [37] was used. The protonation states were calculated, assuming a neutral pH. The PM3 Hamiltonian, as implemented in the MOPAC package (MOPAC2016 v. 18.151, Stewart Computational Chemistry, Colorado Springs, CO, USA) [38,39,40], was then used to further optimize the 3D-structures before the alignment for the docking calculations.

### 3.4. Molecular Docking

Flexible ligand docking experiments were performed by employing AutoDock 4.2.6 and AutoDock Vina software implemented in YASARA (v. 19.5.5, YASARA Biosciences GmbH, Vienna, Austria) [41,42], using the three-dimensional crystal structure of SARS-CoV-2 M^pro^ in complex with an inhibitor N3 PRD_002214 (PDB ID: 6LU7) obtained from the Protein Data Bank (PDB, http://www.rcsb.org/pdb), and the Lamarckian genetic algorithm (LGA). The covalent bond between the Cys145 residue and the crystallized ligand has been eliminated. His41 and Cys145 residues were protonated and optimized using YASARA software. The maps were generated by the program AutoGrid (4.2.6) with a spacing of 0.375 Å and dimensions that encompass all atoms extending 5 Å from the surface of the structure of the crystallized ligand. Point charges were initially assigned according to the AMBER03 force field, and then damped to mimic the less polar Gasteiger charges used to optimize the AutoDock scoring function. All parameters were inserted at their default settings, as previously reported [43]. In the docking tab, the macromolecule and ligand were selected, and GA parameters were set as ga_runs = 100, ga_pop_size = 150, ga_num_evals = 25,000,000, ga_num_generations = 27,000, ga_elitism = 1, ga_mutation_rate = 0.02, ga_crossover_rate = 0.8, ga_crossover_mode = two points, ga_cauchy_alpha = 0.0, ga_cauchy_beta = 1.0, number of generations for picking worst individual = 10.

### 3.5. Molecular Dynamics Simulations

The molecular dynamics simulations of the M^pro^/ligand complexes were performed with the YASARA Structure package. A periodic cubic simulation cell with boundaries extending 8 Å [44] from the surface of the complex was employed. The box was filled with water, with a maximum sum of all water bumps of 1.0 Å, and a density of 0.997 g mL^−1^.

The setup included an optimization of the hydrogen bonding network [45] to increase the solute stability, and a p*K*_a_ prediction to fine-tune the protonation states of protein residues at the chosen pH of 7.4 [46]. NaCl ions were added with a physiological concentration of 0.9%, with an excess of either Na or Cl to neutralize the cell. Water molecules were deleted to readjust the solvent density to 0.997 g/mL. The simulation was run using the ff14SB force field [47] for the solute, GAFF2 [48], and AM1BCC [49] for ligands and TIP3P for water. The cutoff was 10 Å for Van der Waals forces (the default used by AMBER [50]) and no cutoff was applied to electrostatic forces (using the Particle Mesh Ewald algorithm, [51]). The equations of motions were integrated with multiple time steps of 2.5 fs for bonded interactions and 5.0 fs for nonbonded interactions at a temperature of 298 K and a pressure of 1 atm (NPT ensemble) using algorithms described in detail previously [52,53]. The final system dimensions were approximately 80 × 80 × 80 Å^3^. Short MD simulation was run on the solvent only to remove clashes. The entire system was then energy minimized using first a steepest descent minimization to remove conformational stress, followed by a simulated annealing minimization until convergence (<0.01 kcal/mol Å). Finally, 10 ns MD simulations without any restrictions were conducted, and the conformations of each system were recorded every 200 ps. After inspection of the solute RMSD as a function of simulation time, the last 3 ns averaged structures were considered for further analysis.

## 4. Conclusions

Natural compounds can represent a synergy to be combined with pharmacological treatments in various pathologies [54]. In some cases, the natural resource is not readily available and cannot be reproduced on a large scale (for example, marine sponges) to be included in the global market. In other cases, the raw materials for the extraction, purification, or enrichment of bioactive compounds can be reproducible (for example, algae) for the formulation of supplements.

Here, we described the screening of a collection of MNP (14,064 compounds) in search of new, potential SARS-CoV-2 M^pro^ inhibitors. Structure-based and ligand-based drug design approaches have been exploited as valuable drug discovery tools, owing to their versatility and synergistic character [43,55,56].

For the ligand-based evaluation, we used a pharmacophore model developed by the Pharmit server, whereas, for the structure-based evaluation, an initial docking analysis was performed, followed by a parallel docking approach on lead molecules. The poses of the 17 selected ligands have been analyzed by MD simulations. The selected compounds showed a better energy score than the drug currently used to treat COVID-19. Furthermore, it has been shown that several classes of compounds, such as phlorotannins, flavonoids, and pseudo peptides, can inhibit the SARS-CoV-2 M^pro^, as demonstrated for the SARS-CoV-1 M^pro^.

Future in vitro activity assays of the ligands identified in this study will provide vital information on novel scaffolds for lead optimization.

## Figures and Tables

**Table 1:** Structures and calculated free binding energies (Δ*G*_B_, in kcal/mol) of the selected Marine Natural Products (MNP) (compounds **1**–**17**), N3 ligand (**18**), and Lopinavir (**19**).

Compound/Name	MNP ID	Structure	Δ*G*_B_ Vina	Δ*G*_B_ Autodock4	Average Δ*G*_B_	Δ*G*_B_ Re-docking Vina
**1** Heptafuhalol A	76265-30-0	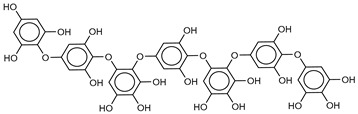	−15.4	−13.8	−14.6	−18.0
**2** Phlorethopentafuhalol B	138551-15-2	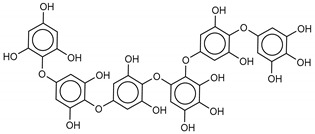	−14.6	−13.9	−14.2	−13.8
**3** Pseudopentafuhalol C	202211-82-3	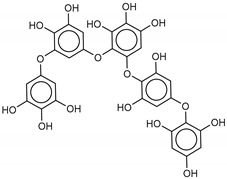	−14.5	−13.8	−14.2	−15.8
**4** Phlorethopentafuhalol A	138529-06-3	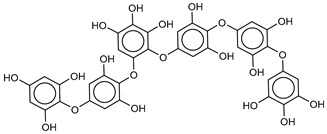	−14.0	−14.3	−14.1	−12.3
**5** Hydroxypentafuhalol A	137809-92-8	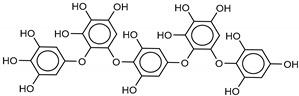	−14.6	−11.7	−13.1	−13.7
**6** Pentaphlorethol B		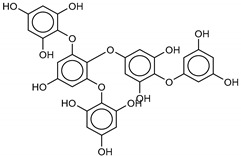	−13.9	−12.2	−13.1	−13.9
**7** 8,8’-Bieckol	89445-12-5	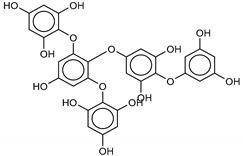	−13.7	−12.1	−12.9	−13.5
**8** Apigenin-7-O-neohesperidoside	36790-49-5	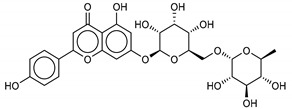	−12.4	−12.3	−12.4	−12.8
**9** Luteolin-7-rutinoside	20633-84-5	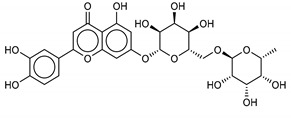	−12.1	−12.3	−12.2	−12.2
**10** 6,6’-Bieckol	88095-81-2	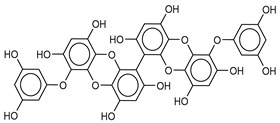	−12.2	−12.0	−12.1	−15.8
**11** Dieckol	88095-77-6	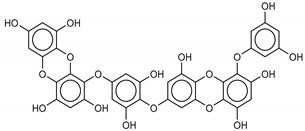	−12.0	−12.1	−12.1	−12.9
**12** Pseudotheonamide D	224577-36-0	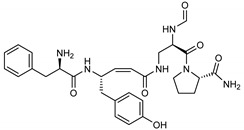	−12.2	−10.9	−11.6	−12.2 (−14.9) ^a^
**13** Aeruginosin 98B	167228-01-5	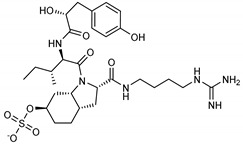	−12.1	−10.4	−11.3	−12.5
**14** Resinoside B	144027-79-2	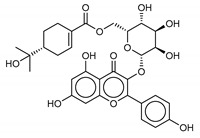	−12.2	−10.2	−11.2	−11.4
**15** Pentaphlorethol A	164176-23-2	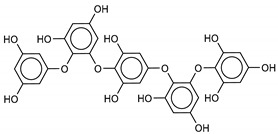	−12.8	−9.4	−11.1	−14.5
**16** Tunichrome An2	115982-31-5	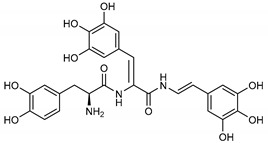	−11.5	−10.5	−11.0	−13.5
**17** Pseudotheonamide C	224577-35-9	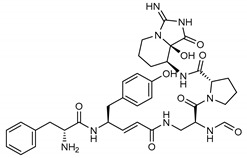	−10.5	−10.9	−10.7	−11.0 (−14.4) ^a^
**18** *N*-[(5-methylisoxazol-3-yl)carbonyl]alanyl-l-valyl-*N*^1^-((1*R*,2*Z*)-4-(benzyloxy)-4-oxo-1-{[(3*R*)-2-oxopyrrolidin-3-yl]methyl}but-2-enyl)-l-leucinamide	PRD_002214	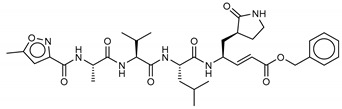	−11.9 (−15.0) ^a^	−11.0 (−15.3) ^a^	−11.4 (−15.1) ^a^	−14.5 (−15.1) ^a^
**19** Lopinavir	92727	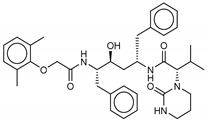	−10.3	−10.3	−10.3	−12.5

^a^ From covalent docking.

## Supplementary Materials

Supplementary materials can be found at https://www.mdpi.com/1660-3397/18/4/225/s1.
