# Supplementary material for: Putative Inhibitors of SARS-CoV-2 Main Protease from A Library of Marine Natural Products: A Virtual Screening and Molecular Modeling Study
Source: Mar Drugs. 2020 Apr 23;18(4):225. doi: 10.3390/md18040225 (PMC7231030; doi:10.3390/md18040225)
Supplement: Supplementary file 1 [file marinedrugs-18-00225-s001.pdf]

marine drugs

Supporting Information

Putative Inhibitors of SARS-CoV-2 Main Protease from A Library of Marine Natural Products: A Virtual Screening and Molecular Modeling Study

Davide Gentile [1,*], Vincenzo Patamia [1], Angela Scala [2], Maria Teresa Sciortino [2], Anna Piperno [2] and Antonio Rescifina [1,3,*]

[1] Department of Drug Sciences, University of Catania, V.le A. Doria, Catania 95125, Italy; vincenzo.patamia@unict.it
[2] Department of Chemical, Biological, Pharmaceutical, and Environmental Sciences, University of Messina, V.le F. Stagno d'Alcontres 31, Messina 98166, Italy; ascala@unime.it (A.S.); mtsciortino@unime.it (M.T.S.); apiperno@unime.it (A.P.)
[3] Consorzio Interuniversitario Nazionale di ricerca in Metodologie e Processi Innovativi di Sintesi (CINMPS), Via E. Orabona, 4, Bari 70125, Italy
* Correspondence: davide.gentile@unict.it (D.G.); arescifina@unict.it (A.R.); Tel.: +39-095-738-5017

Table of content

Figure S1. Total energy, RMSDs, ligand interaction and docking binding pose of 76265-30-0 S2
Figure S2. Total energy, RMSDs, ligand interaction and docking binding pose of 138551-15-2 S2
Figure S3. Total energy, RMSDs, ligand interaction and docking binding pose of 202211-82-3 S3
Figure S4. Total energy, RMSDs, ligand interaction and docking binding pose of 138529-06-3 S3
Figure S5. Total energy, RMSDs, ligand interaction and docking binding pose of 137809-92-8 S4
Figure S6. Total energy, RMSDs, ligand interaction and docking binding pose of Pentaphlorethol B S4
Figure S7. Total energy, RMSDs, ligand interaction and docking binding pose of 89445-12-5 S5
Figure S8. Total energy, RMSDs, ligand interaction and docking binding pose of 36790-49-5 S5
Figure S9. Total energy, RMSDs, ligand interaction and docking binding pose of 20633-84-5 S6
Figure S10. Total energy, RMSDs, ligand interaction and docking binding pose of 88095-81-2 S6
Figure S11. Total energy, RMSDs, ligand interaction and docking binding pose of 88095-77-6 S7
Figure S12. Total energy, RMSDs, ligand interaction and docking binding pose of 224577-36-0 S7
Figure S13. Total energy, RMSDs, ligand interaction and docking binding pose of 167228-01-5 S8
Figure S14. Total energy, RMSDs, ligand interaction and docking binding pose of 144027-79-2 S8
Figure S15. Total energy, RMSDs, ligand interaction and docking binding pose of 164176-23-2 S9
Figure S16. Total energy, RMSDs, ligand interaction and docking binding pose of 115982-31-5 S9
Figure S17. Total energy, RMSDs, ligand interaction and docking binding pose of 224577-35-9 S10
Figure S18. Total energy, RMSDs, ligand interaction and docking binding pose of N3 S10
Figure S19. Total energy, RMSDs, ligand interaction and docking binding pose of 92727 S11
Table S1. Virtual screening of chemical structures of the marine dataset that passed the pharmacophore filter including the contacting receptor residues and binding energy S11

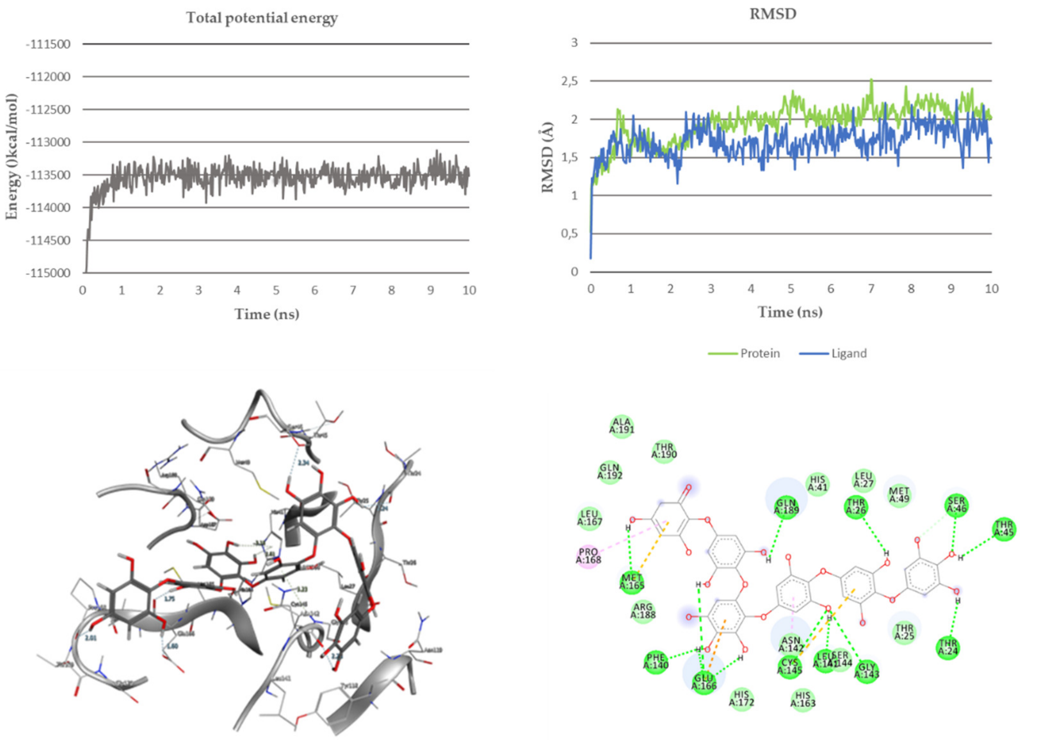

Figure S1. MNP ID 76265-30-0. Total energy (up-left) and RMSDs (up-right) of Mpro enzyme and its complexes with ligand. Docking binding pose (down-left) and Interaction (down-right) inside the binding pocket of Mpro enzyme.

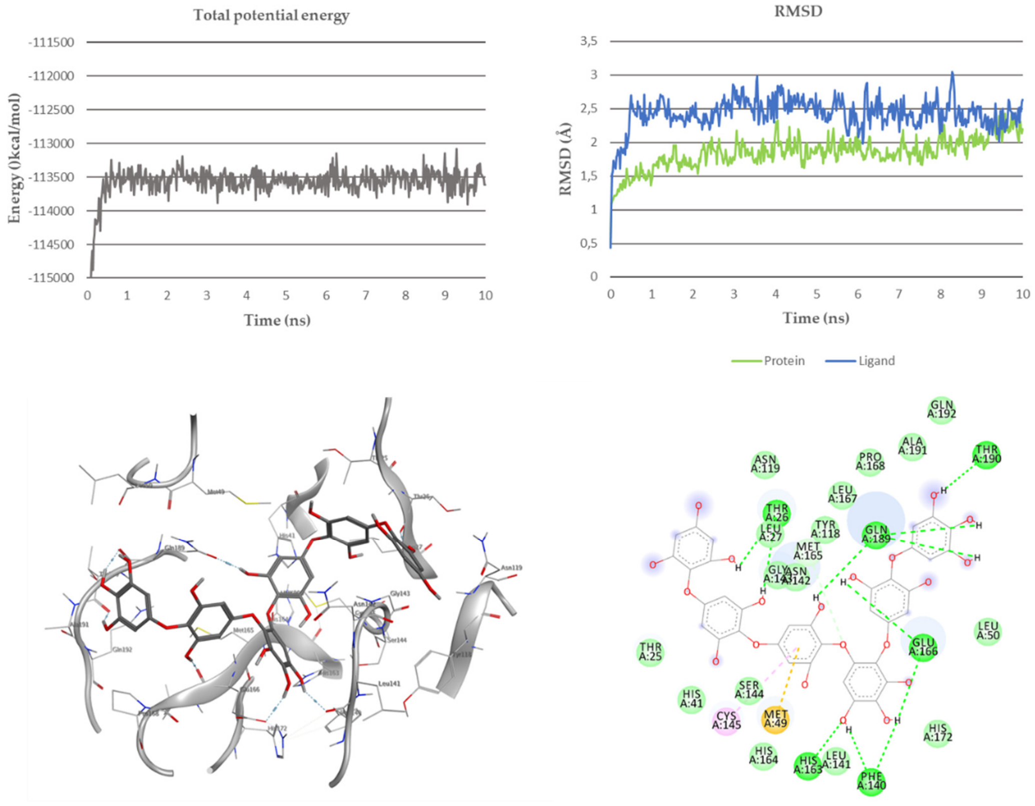

Figure S2. MNP ID 138551-15-2. Total energy (up-left) and RMSDs (up-right) of Mpro enzyme and its complexes with ligand. Docking binding pose (down-left) and Interaction (down-right) inside the binding pocket of Mpro enzyme.

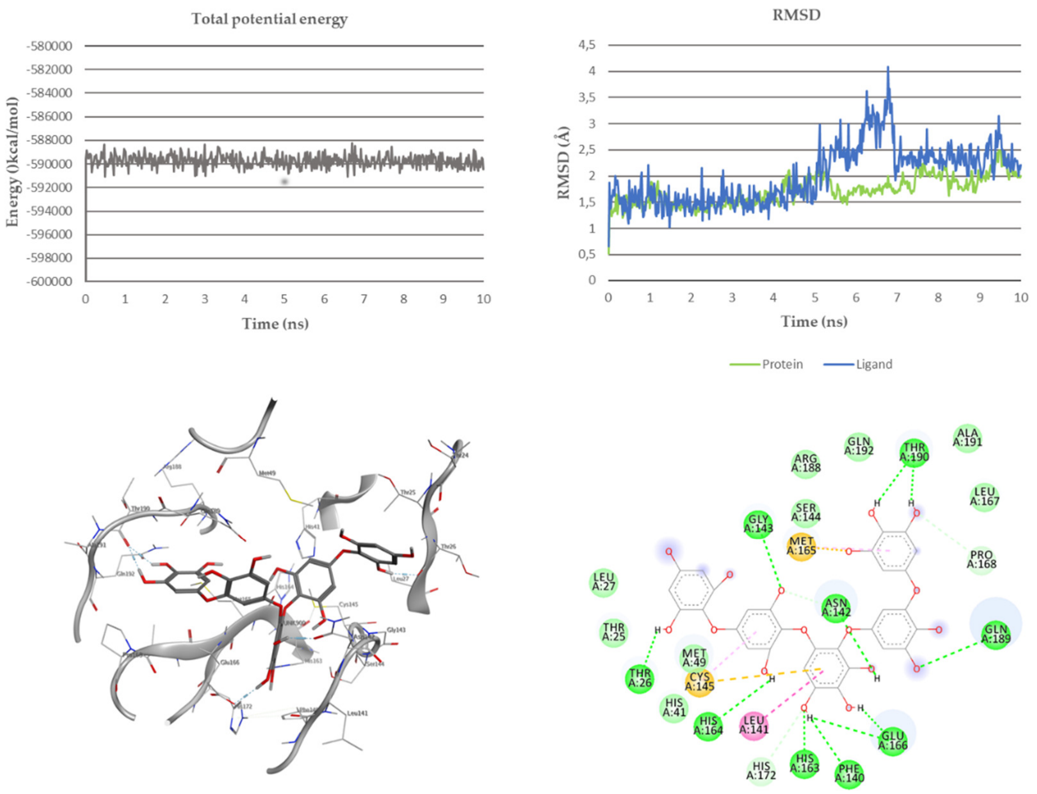

Figure S3. MNP ID 202211-82-3. Total energy (up-left) and RMSDs (up-right) of M^{pro} enzyme and its complexes with ligand. Docking binding pose (down-left) and Interaction (down-right) inside the binding pocket of M^{pro} enzyme.

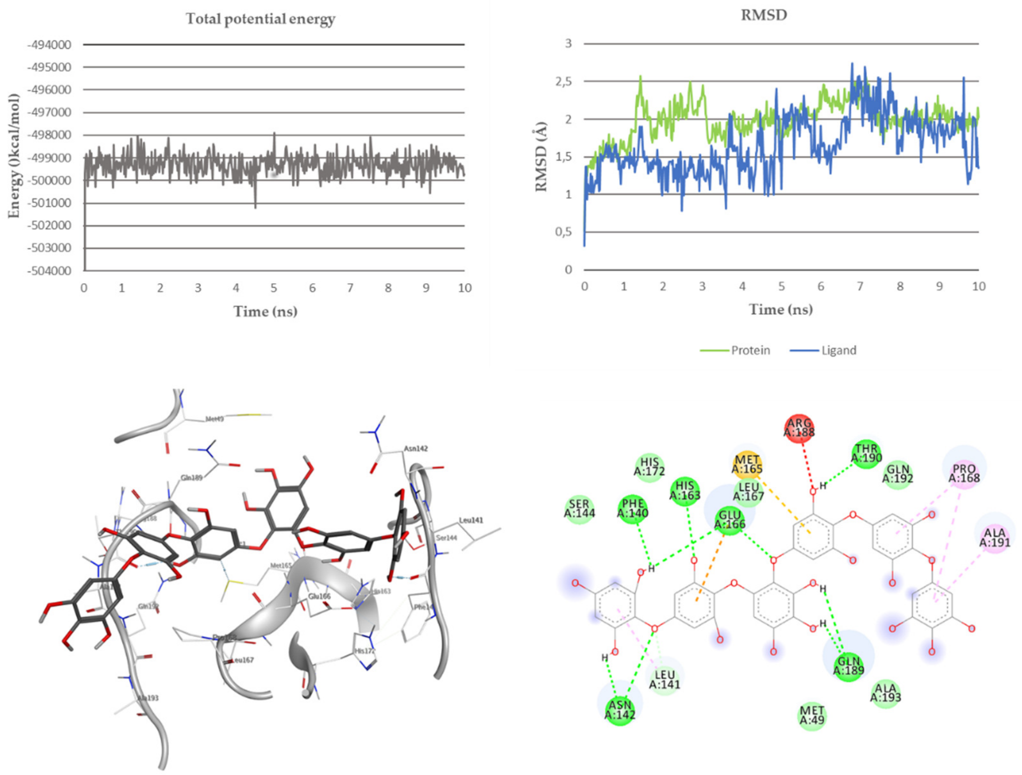

Figure S4. MNP ID 138529-06-3. Total energy (up-left) and RMSDs (up-right) of M^{pro} enzyme and its complexes with ligand. Docking binding pose (down-left) and Interaction (down-right) inside the binding pocket of M^{pro} enzyme.

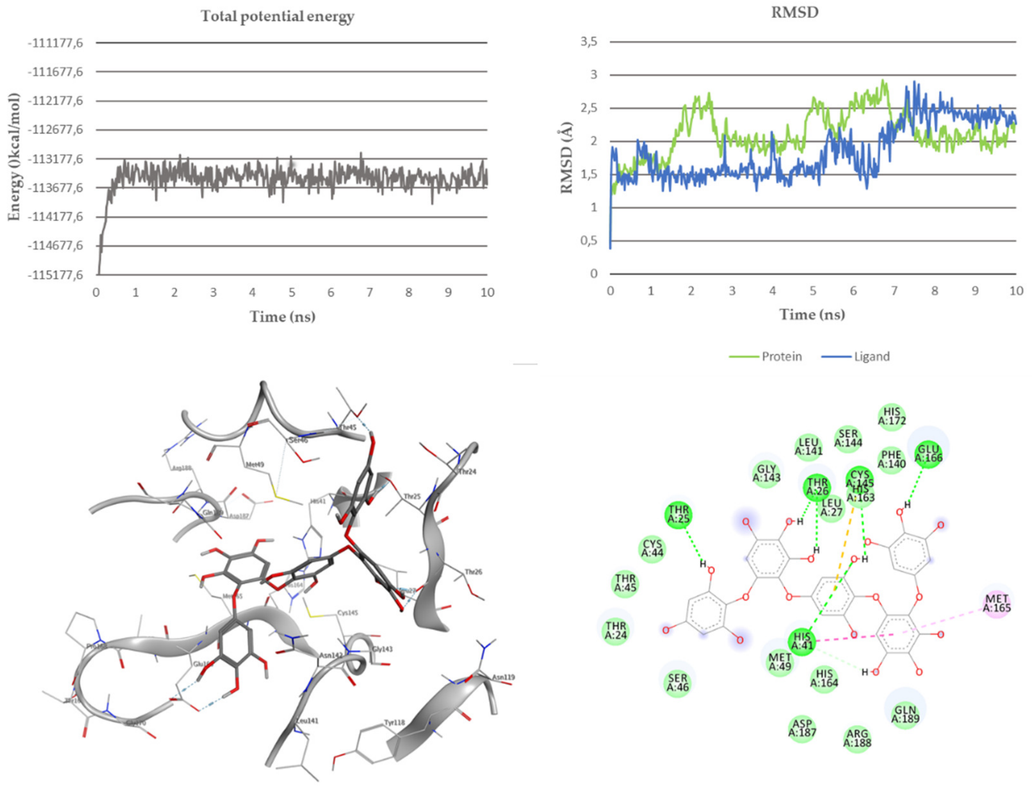

Figure S5. MNP ID 137809-92-8. Total energy (up-left) and RMSDs (up-right) of M^{pro} enzyme and its complexes with ligand. Docking binding pose (down-left) and Interaction (down-right) inside the binding pocket of M^{pro} enzyme.

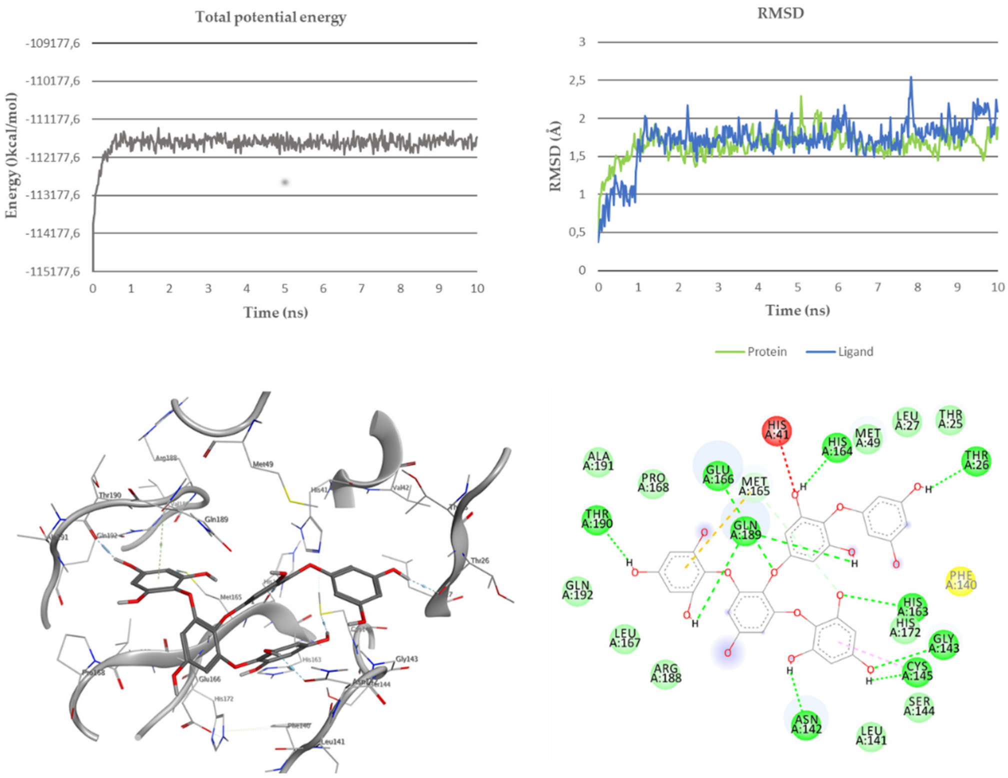

Figure S6. MNP ID Pentaphlorethol B. Total energy (up-left) and RMSDs (up-right) of M^{pro} enzyme and its complexes with ligand. Docking binding pose (down-left) and Interaction (down-right) inside the binding pocket of M^{pro} enzyme.

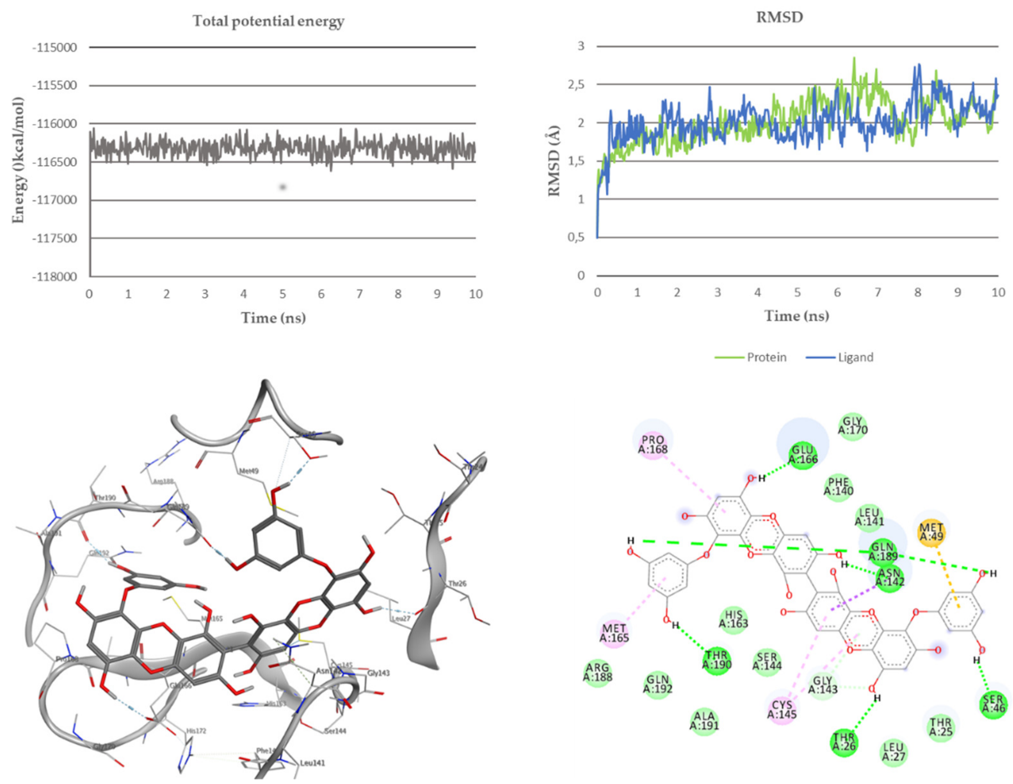

Figure S7. MNP ID 89445-12-5. Total energy (up-left) and RMSDs (up-right) of Mpro enzyme and its complexes with ligand. Docking binding pose (down-left) and Interaction (down-right) inside the binding pocket of Mpro enzyme.

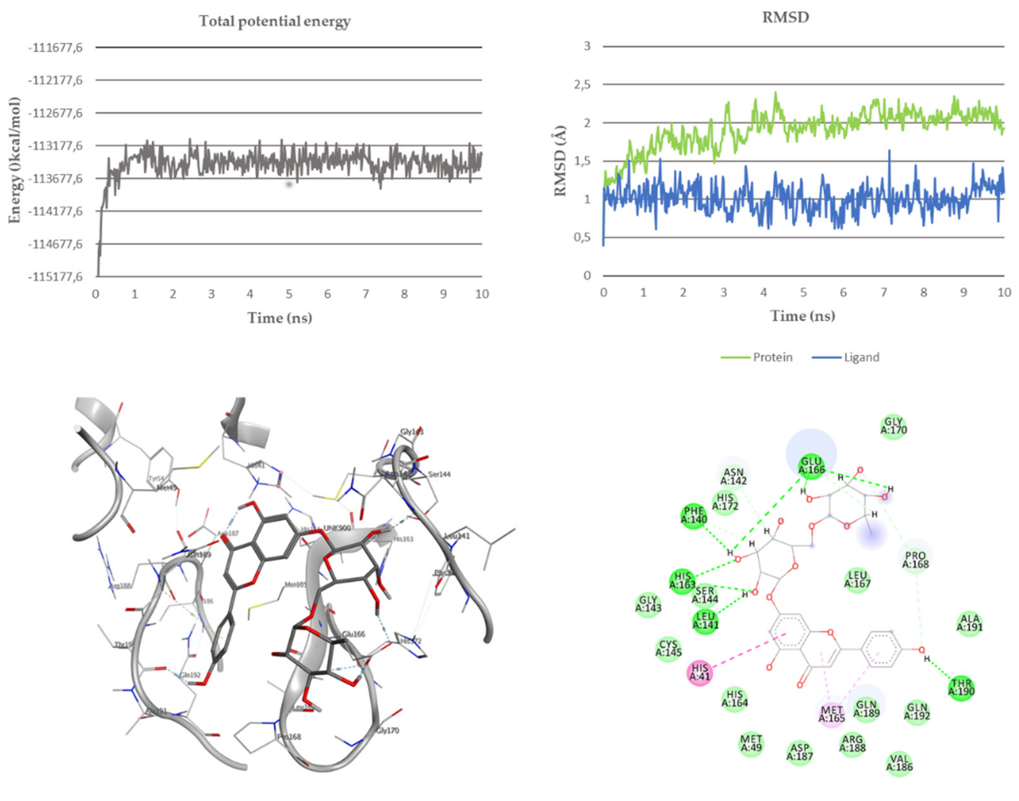

Figure S8. MNP ID 36790-49-5. Total energy (up-left) and RMSDs (up-right) of Mpro enzyme and its complexes with ligand. Docking binding pose (down-left) and Interaction (down-right) inside the binding pocket of Mpro enzyme.

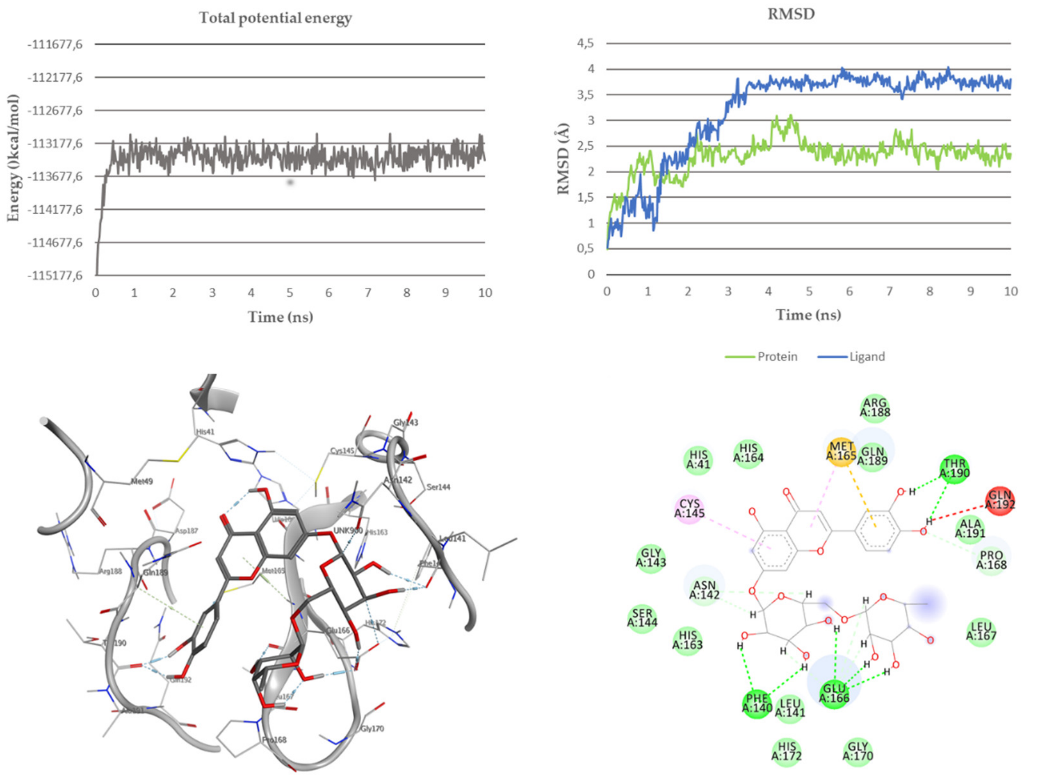

Figure S9. MNP ID 20633-84-5. Total energy (up-left) and RMSDs (up-right) of M^{pro} enzyme and its complexes with ligand. Docking binding pose (down-left) and Interaction (down-right) inside the binding pocket of M^{pro} enzyme.

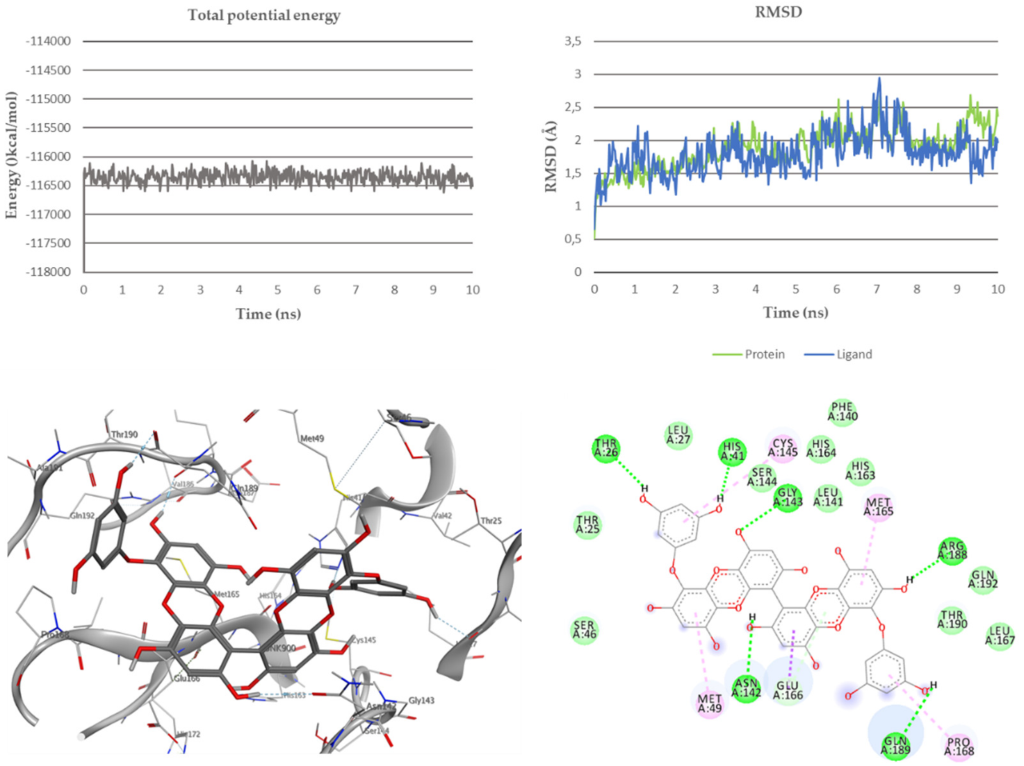

Figure S10. MNP ID 88095-81-2. Total energy (up-left) and RMSDs (up-right) of M^{pro} enzyme and its complexes with ligand. Docking binding pose (down-left) and Interaction (down-right) inside the binding pocket of M^{pro} enzyme.

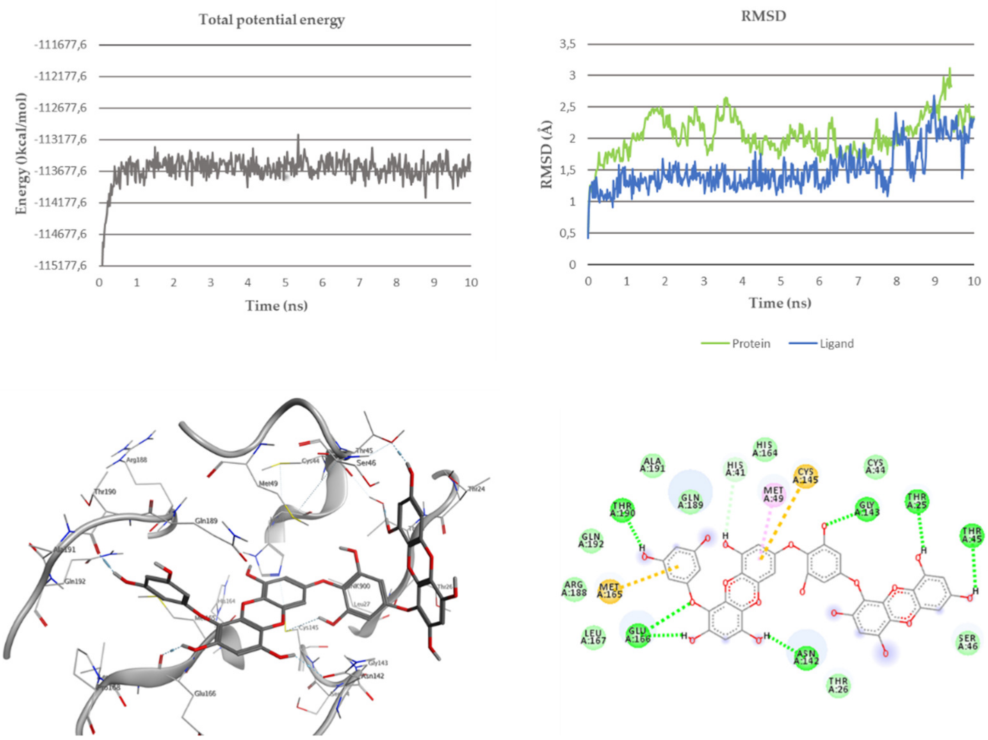

Figure S11. MNP ID 88095-81-2. Total energy (up-left) and RMSDs (up-right) of M^{pro} enzyme and its complexes with ligand. Docking binding pose (down-left) and Interaction (down-right) inside the binding pocket of M^{pro} enzyme.

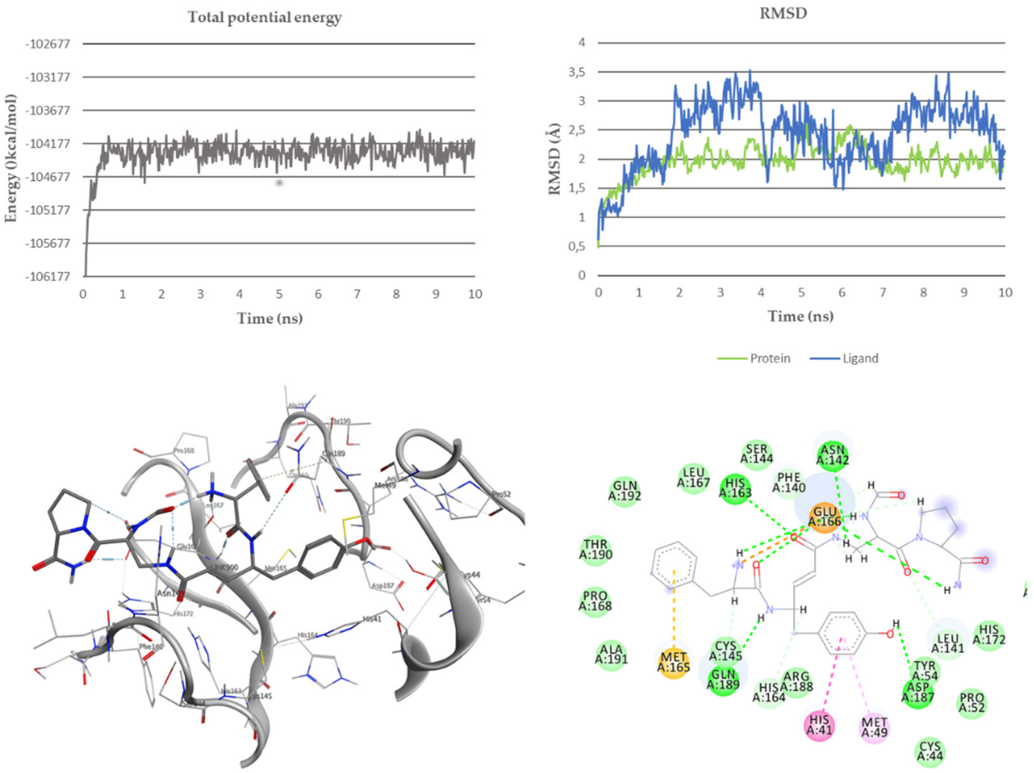

Figure S12. MNP ID 224577-36-0. Total energy (up-left) and RMSDs (up-right) of M^{pro} enzyme and its complexes with ligand. Docking binding pose (down-left) and Interaction (down-right) inside the binding pocket of M^{pro} enzyme.

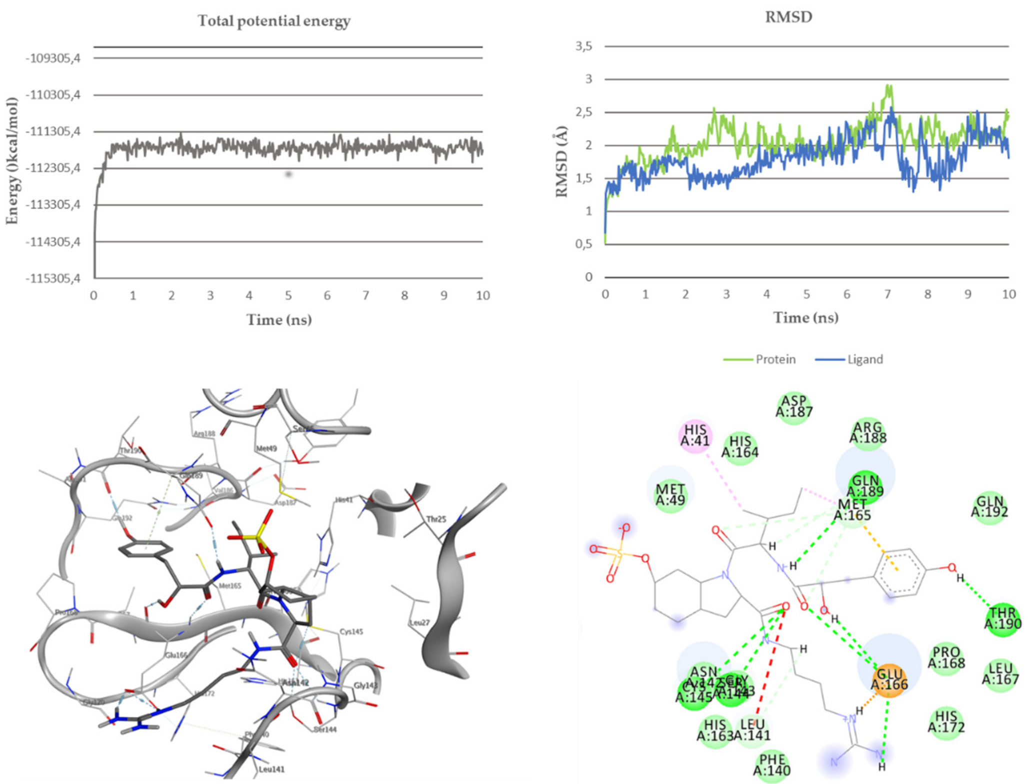

Figure S13. MNP ID 167228-01-5. Total energy (up-left) and RMSDs (up-right) of Mpro enzyme and its complexes with ligand. Docking binding pose (down-left) and Interaction (down-right) inside the binding pocket of Mpro enzyme.

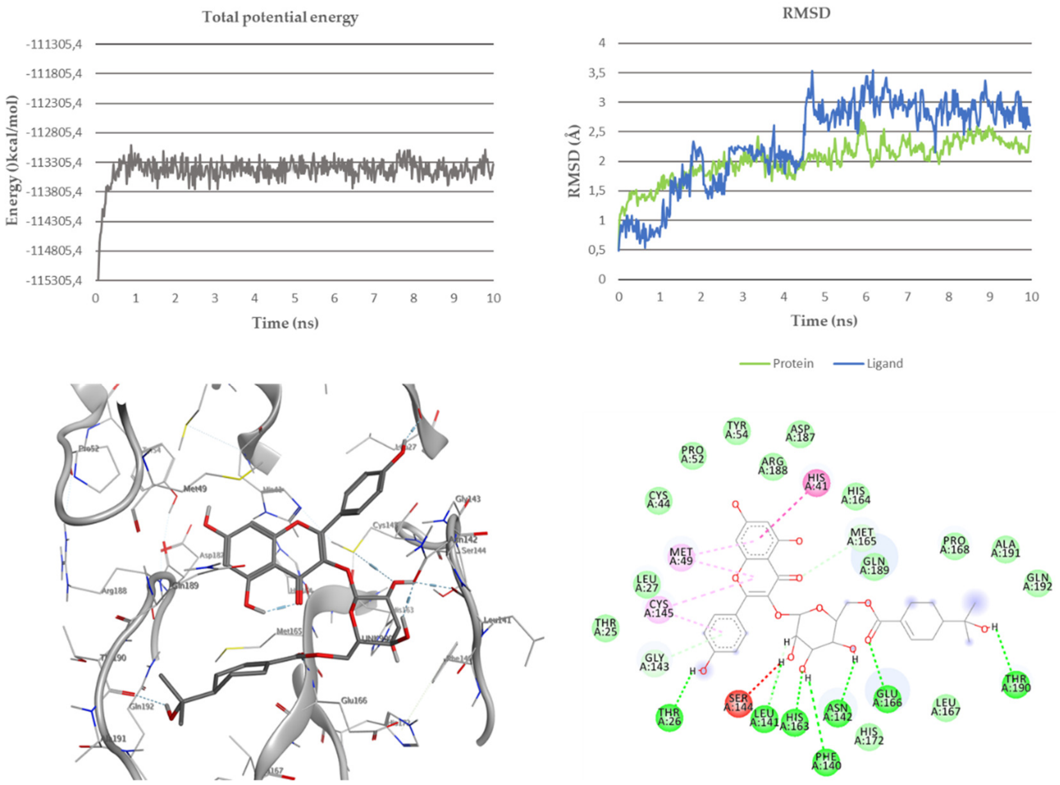

Figure S14. MNP ID 144027-79-2. Total energy (up-left) and RMSDs (up-right) of Mpro enzyme and its complexes with ligand. Docking binding pose (down-left) and Interaction (down-right) inside the binding pocket of Mpro enzyme.

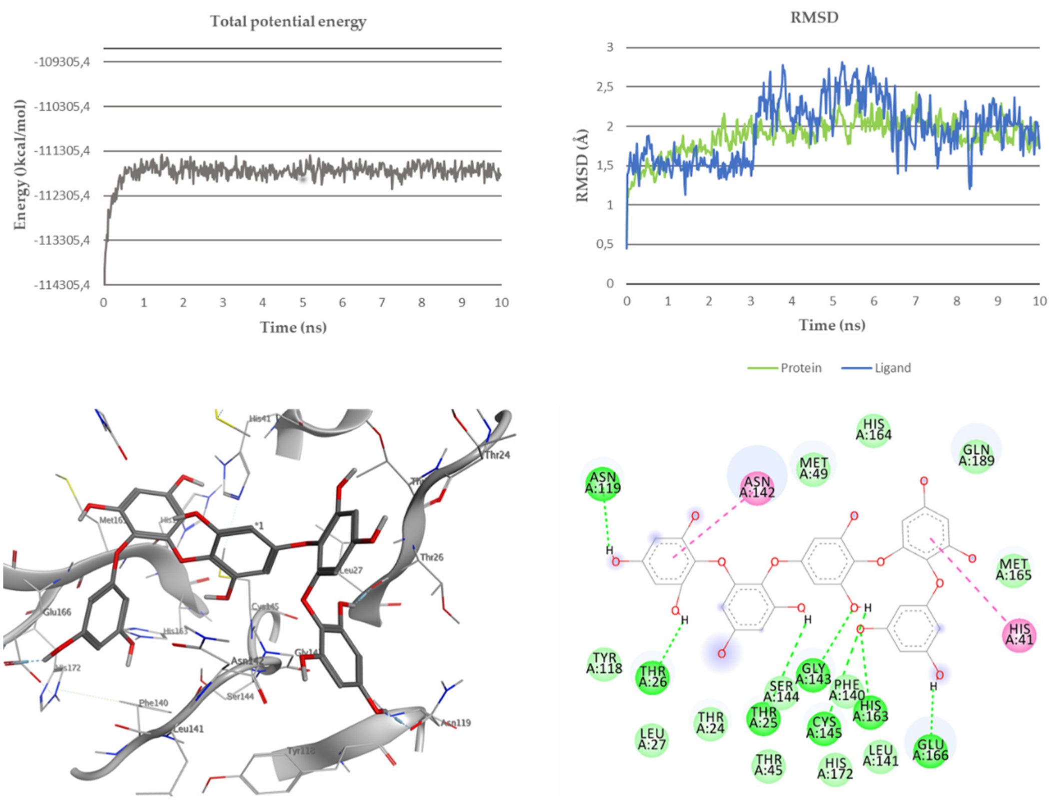

Figure S15. MNP ID 164176-23-2. Total energy (up-left) and RMSDs (up-right) of M^pro enzyme and its complexes with ligand. Docking binding pose (down-left) and Interaction (down-right) inside the binding pocket of M^pro enzyme.

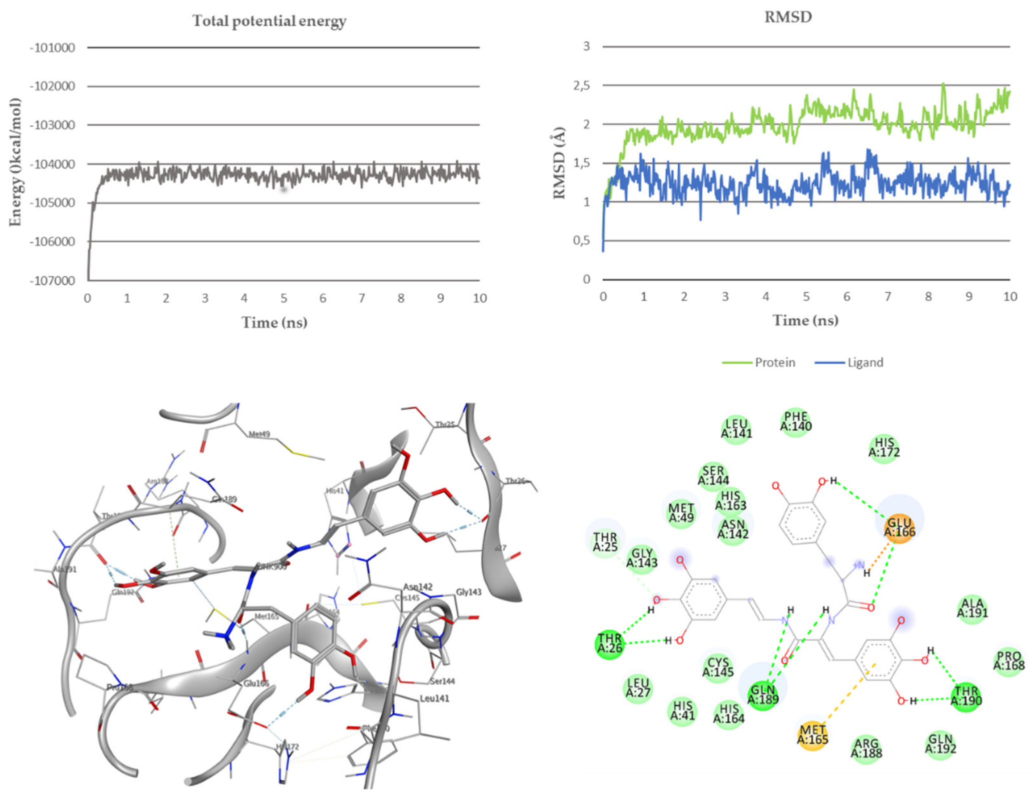

Figure S16. MNP ID 115982-31-5. Total energy (up-left) and RMSDs (up-right) of M^pro enzyme and its complexes with ligand. Docking binding pose (down-left) and Interaction (down-right) inside the binding pocket of M^pro enzyme.

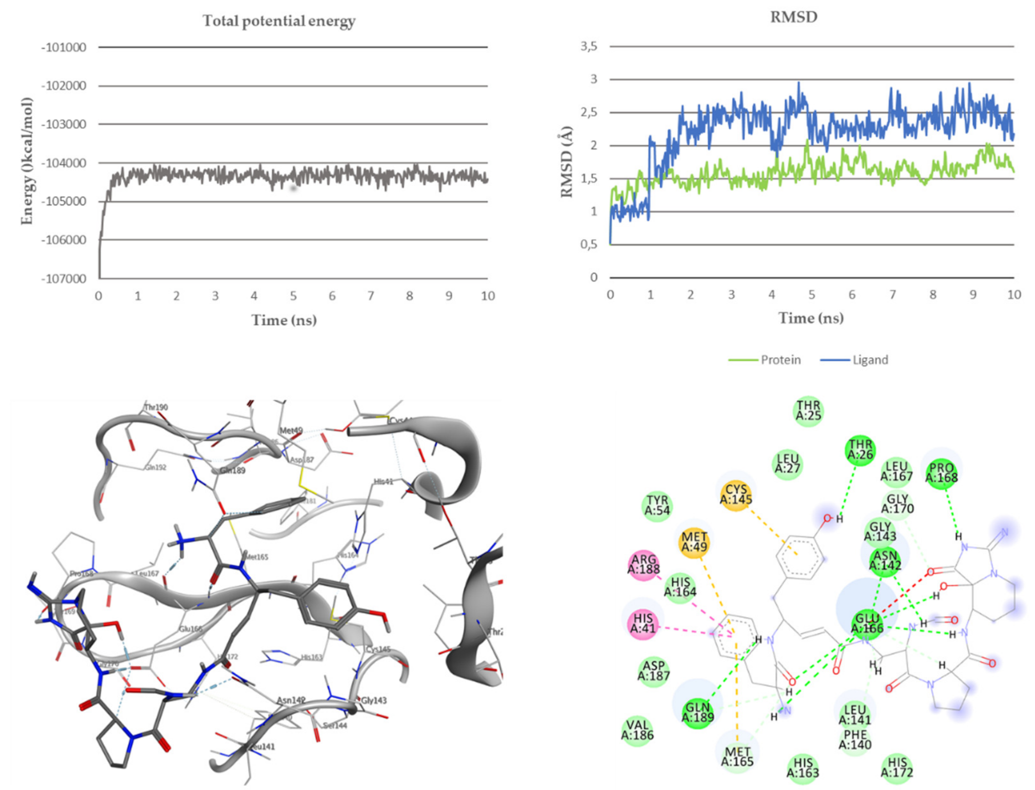

Figure S17. MNP ID 224577-35-9. Total energy (up-left) and RMSDs (up-right) of M^{pro} enzyme and its complexes with ligand. Docking binding pose (down-left) and Interaction (down-right) inside the binding pocket of M^{pro} enzyme.

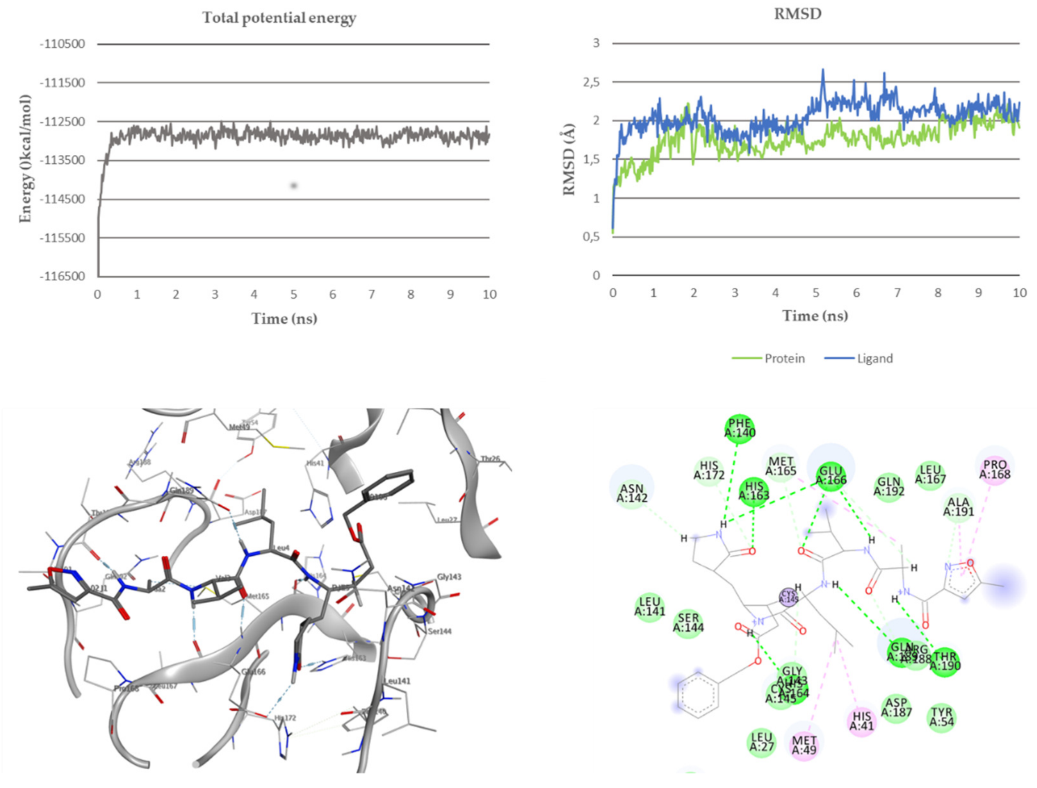

Figure S18. PRD_002214. Total energy (up-left) and RMSDs (up-right) of M^{pro} enzyme and its complexes with ligand. Docking binding pose (down-left) and Interaction (down-right) inside the binding pocket of M^{pro} enzyme.

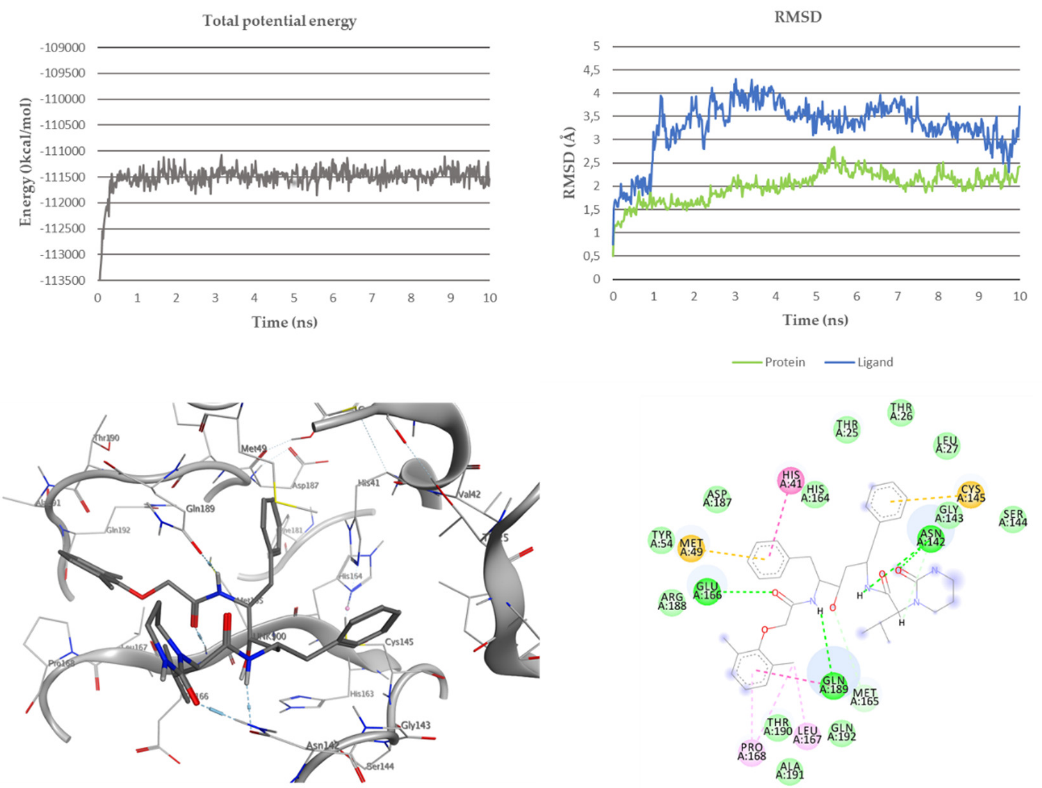

Figure S19. MNP ID 224577-35-9. Total energy (up-left) and RMSDs (up-right) of M^pro enzyme and its complexes with ligand. Docking binding pose (down-left) and Interaction (down-right) inside the binding pocket of M^pro enzyme.

Table S1. Virtual screening of chemical structures of the marine dataset that passed the pharmacophore filter including the contacting receptor residues and binding energy.

MNP ID[a]	Contacting receptor residues	Binding Energy (kcal/mol) Vina
78798-08-0	A THR 25 A THR 26 A LEU 27 A MET 49 A PHE 140 A LEU 141 A ASN 142 A GLY 143 A SER 144 A CYS 145 A HIS 163 A HIS 164 A MET 165 A GLU 166 A LEU 167 A PRO 168 A HIS 172 A ARG 188 A GLN 189 A THR 190 A ALA 191 A GLN 192	−10.18
211686-51-0	A THR 24 A THR 25 A THR 26 A HIS 41 A MET 49 A TYR 118 A ASN 119 A PHE 140 A LEU 141 A ASN 142 A GLY 143 A SER 144 A CYS 145 A HIS 163 A MET 165 A GLU 166 A LEU 167 A PRO 168 A HIS 172 A GLN 189 A THR 190 A ALA 191	−10.00
40663-80-7	A THR 24 A THR 25 A THR 26 A LEU 27 A HIS 41 A THR 45 A SER 46 A MET 49 A PHE 140 A LEU 141 A ASN 142 A GLY 143 A SER 144 A CYS 145 A HIS 163 A HIS 164 A MET 165 A GLU 166 A LEU 167 A PRO 168 A HIS 172 A GLN 189 A THR 190 A ALA 191 A GLN 192	−9.95
1008752-06-	A PHE 140 A LEU 141 A ASN 142 A GLY 143 A SER 144 A CYS 145 A HIS 163 A HIS 164 A MET 165 A GLU 166 A LEU 167 A PRO 168 A GLN 189 A THR 190	−9.62
104196-68-1	A HIS 41 A TYR 54 A PHE 140 A LEU 141 A ASN 142 A SER 144 A CYS 145 A HIS 163 A HIS 164 A MET 165 A GLU 166 A PRO 168 A HIS 172 A ASP 187 A ARG 188 A GLN 189 A THR 190 A GLN 192	−9.61

07900-75-4	A PHE 140 A LEU 141 A ASN 142 A GLY 143 A SER 144 A CYS 145 A HIS 163 A HIS 164 A MET 165 A GLU 166 A LEU 167 A PRO 168 A GLN 189 A THR 190	−9.61
144027-79-2	A THR 25 A THR 26 A LEU 27 A HIS 41 A MET 49 A TYR 54 A PHE 140 A LEU 141 A ASN 142 A GLY 143 A SER 144 A CYS 145 A HIS 163 A MET 165 A GLU 166 A LEU 167 A PRO 168 A HIS 172 A ASP 187 A ARG 188 A GLN 189 A THR 190 A ALA 191 A GLN 192	−9.56
858950-40-0	A THR 25 A THR 26 A LEU 27 A HIS 41 A MET 49 A ASN 142 A GLY 143 A SER 144 A CYS 145 A HIS 163 A HIS 164 A MET 165 A GLU 166 A LEU 167 A PRO 168 A ARG 188 A GLN 189 A THR 190 A ALA 191 A GLN 192	−9.56
18115-90-7	A MET 49 A PHE 140 A LEU 141 A ASN 142 A GLY 143 A SER 144 A CYS 145 A HIS 163 A HIS 164 A MET 165 A GLU 166 A LEU 167 A PRO 168 A HIS 172 A ARG 188 A GLN 189	−9.43
152845-74-4	A THR 25 A THR 26 A LEU 27 A HIS 41 A MET 49 A LEU 141 A ASN 142 A GLY 143 A CYS 145 A MET 165 A GLU 166 A PRO 168 A ARG 188 A GLN 189 A THR 190 A GLN 192	−9.35
58950-46-6	A THR 25 A THR 26 A LEU 27 A HIS 41 A MET 49 A LEU 141 A ASN 142 A GLY 143 A SER 144 A CYS 145 A HIS 164 A MET 165 A GLU 166 A LEU 167 A PRO 168 A ARG 188 A GLN 189 A THR 190	−9.31
147716-81-2	A THR 24 A THR 25 A THR 26 A LEU 27 A HIS 41 A THR 45 A SER 46 A MET 49 A PHE 140 A LEU 141 A ASN 142 A GLY 143 A SER 144 A CYS 145 A HIS 163 A MET 165 A GLU 166 A HIS 172 A GLN 189	−9.29
151379-39-4	A THR 24 A THR 25 A THR 26 A LEU 27 A HIS 41 A THR 45 A SER 46 A MET 49 A PHE 140 A LEU 141 A ASN 142 A GLY 143 A SER 144 A CYS 145 A HIS 163 A MET 165 A GLU 166 A HIS 172 A GLN 189	−9.28
79849-37-9	A THR 25 A THR 26 A LEU 27 A HIS 41 A VAL 42 A MET 49 A ASN 142 A GLY 143 A SER 144 A CYS 145 A HIS 164 A MET 165 A GLU 166 A LEU 167 A PRO 168 A ARG 188 A GLN 189 A THR 190 A ALA 191 A GLN 192	−9.22
134458-00-7	A THR 25 A LEU 27 A HIS 41 A MET 49 A ASN 142 A GLY 143 A CYS 145 A HIS 164 A MET 165 A GLU 166 A LEU 167 A PRO 168 A ARG 188 A GLN 189 A THR 190 A GLN 192	−9.22
65745-47-3	A THR 24 A THR 25 A THR 26 A LEU 27 A HIS 41 A SER 46 A MET 49 A PHE 140 A LEU 141 A ASN 142 A GLY 143 A SER 144 A CYS 145 A HIS 163 A HIS 164 A MET 165 A GLU 166 A HIS 172 A GLN 189	−9.14
514830-04-7	A THR 25 A LEU 27 A HIS 41 A MET 49 A PHE 140 A LEU 141 A ASN 142 A GLY 143 A SER 144 A CYS 145 A HIS 163 A HIS 164 A MET 165 A GLU 166 A PRO 168 A HIS 172 A ASP 187 A ARG 188 A GLN 189 A THR 190 A ALA 191	−9.14
113540-81-1	A THR 25 A LEU 27 A HIS 41 A MET 49 A PHE 140 A LEU 141 A ASN 142 A GLY 143 A SER 144 A CYS 145 A HIS 163 A HIS 164 A MET 165 A GLU 166 A PRO 168 A HIS 172 A ASP 187 A ARG 188 A GLN 189 A THR 190 A ALA 191	−9.13
97888-14-7	A HIS 41 A CYS 44 A MET 49 A PRO 52 A TYR 54 A PHE 140 A LEU 141 A ASN 142 A GLY 143 A SER 144 A CYS 145 A HIS 164 A MET 165 A GLU 166 A LEU 167 A PRO 168 A VAL 186 A ASP 187 A ARG 188 A GLN 189 A THR 190 A ALA 191 A GLN 192	−9.00
144587-57-5	A THR 25 A THR 26 A LEU 27 A HIS 41 A MET 49 A PHE 140 A LEU 141 A ASN 142 A GLY 143 A SER 144 A CYS 145 A HIS 163 A HIS 164 A MET 165 A GLU 166 A PRO 168 A GLN 189 A THR 190 A ALA 191	−8.94

757976-67-3	A THR 24 A THR 25 A THR 26 A LEU 27 A HIS 41 A CYS 44 A THR 45 A SER 46 A MET 49 A TYR 54 A ASN 142 A GLY 143 A CYS 145 A HIS 163 A HIS 164 A MET 165 A GLU 166 A ASP 187 A ARG 188 A GLN 189	−8.92
97948-90-8	A HIS 41 A CYS 44 A MET 49 A PRO 52 A TYR 54 A PHE 140 A LEU 141 A ASN 142 A GLY 143 A SER 144 A CYS 145 A HIS 164 A MET 165 A GLU 166 A LEU 167 A PRO 168 A ASP 187 A ARG 188 A GLN 189 A THR 190 A GLN 192	−8.91
89444-89-3	A THR 25 A THR 26 A LEU 27 A HIS 41 A MET 49 A PHE 140 A LEU 141 A ASN 142 A SER 144 A CYS 145 A HIS 163 A HIS 164 A MET 165 A GLU 166 A PRO 168 A ASP 187 A ARG 188 A GLN 189 A THR 190 A GLN 192	−8.91
97888-13-6	A HIS 41 A CYS 44 A MET 49 A PRO 52 A TYR 54 A PHE 140 A LEU 141 A ASN 142 A GLY 143 A SER 144 A CYS 145 A HIS 164 A MET 165 A GLU 166 A LEU 167 A PRO 168 A ASP 187 A ARG 188 A GLN 189 A THR 190 A GLN 192	−8.91
131727-01-0	A LEU 50 A PHE 140 A LEU 141 A ASN 142 A HIS 163 A MET 165 A GLU 166 A LEU 167 A PRO 168 A HIS 172 A ARG 188 A GLN 189 A THR 190 A ALA 191 A GLN 192	−8.87
175669-27-9	A HIS 41 A MET 49 A TYR 54 A PHE 140 A LEU 141 A ASN 142 A GLY 143 A CYS 145 A HIS 163 A HIS 164 A MET 165 A GLU 166 A LEU 167 A PRO 168 A HIS 172 A ASP 187 A ARG 188 A GLN 189 A THR 190 A ALA 191	−8.85
24577-34-8	A HIS 41 A CYS 44 A MET 49 A PRO 52 A TYR 54 A PHE 140 A LEU 141 A ASN 142 A CYS 145 A HIS 164 A MET 165 A GLU 166 A LEU 167 A PRO 168 A THR 169 A GLY 170 A ASP 187 A ARG 188 A GLN 189 A THR 190 A ALA 191 A GLN 192	−8.84
194427-76-4	A THR 25 A THR 26 A LEU 27 A HIS 41 A MET 49 A PHE 140 A LEU 141 A ASN 142 A GLY 143 A SER 144 A CYS 145 A HIS 163 A HIS 164 A MET 165 A GLU 166 A PRO 168 A HIS 172 A GLN 189 A THR 190 A ALA 191	−8.84
144027-78-1	A THR 25 A THR 26 A LEU 27 A HIS 41 A MET 49 A TYR 54 A ASN 142 A GLY 143 A SER 144 A CYS 145 A HIS 164 A MET 165 A GLU 166 A LEU 167 A PRO 168 A ASP 187 A ARG 188 A GLN 189 A THR 190 A ALA 191 A GLN 192	−8.82
133056-07-2	A THR 25 A THR 26 A LEU 27 A HIS 41 A VAL 42 A MET 49 A LEU 141 A ASN 142 A GLY 143 A SER 144 A CYS 145 A HIS 163 A HIS 164 A MET 165 A GLU 166 A PRO 168 A HIS 172 A ARG 188 A GLN 189 A THR 190 A ALA 191 A GLN 192	−8.81
105239-70-1	A THR 24 A THR 25 A THR 26 A LEU 27 A HIS 41 A CYS 44 A THR 45 A SER 46 A MET 49 A PRO 52 A TYR 54 A LEU 141 A ASN 142 A GLY 143 A SER 144 A CYS 145 A HIS 163 A HIS 164 A MET 165 A GLU 166 A LEU 167 A PRO 168 A HIS 172 A ASP 187 A ARG 188 A GLN 189	−8.81
345642-84-4	A THR 25 A THR 26 A LEU 27 A HIS 41 A MET 49 A PHE 140 A LEU 141 A ASN 142 A GLY 143 A SER 144 A CYS 145 A HIS 163 A MET 165 A GLU 166 A LEU 167 A PRO 168 A ARG 188 A GLN 189 A THR 190 A GLN 192	−8.80
52645-09-7	A THR 25 A THR 26 A LEU 27 A HIS 41 A MET 49 A PHE 140 A LEU 141 A ASN 142 A GLY 143 A SER 144 A CYS 145 A HIS 163 A MET 165 A GLU 166 A LEU 167 A PRO 168 A ARG 188 A GLN 189 A THR 190 A GLN 192	−8.79

105815-90-5	A HIS 41 A PHE 140 A LEU 141 A ASN 142 A GLY 143 A SER 144 A CYS 145 A HIS 163 A HIS 164 A MET 165 A GLU 166 A PRO 168 A HIS 172 A VAL 186 A ASP 187 A ARG 188 A GLN 189 A THR 190	−8.73
17991-67-2	A THR 24 A THR 25 A THR 26 A LEU 27 A MET 49 A PHE 140 A LEU 141 A ASN 142 A GLY 143 A SER 144 A CYS 145 A HIS 163 A HIS 164 A MET 165 A GLU 166 A HIS 172 A GLN 189	−8.72
224577-31-5	A HIS 41 A CYS 44 A MET 49 A PRO 52 A TYR 54 A PHE 140 A LEU 141 A ASN 142 A GLY 143 A CYS 145 A HIS 163 A HIS 164 A MET 165 A GLU 166 A LEU 167 A PRO 168 A THR 169 A GLY 170 A ASP 187 A ARG 188 A GLN 189 A THR 190 A ALA 191 A GLN 192	−8.72
224577-33-7	A HIS 41 A CYS 44 A MET 49 A PRO 52 A TYR 54 A PHE 140 A LEU 141 A ASN 142 A GLY 143 A CYS 145 A HIS 163 A HIS 164 A MET 165 A GLU 166 A LEU 167 A PRO 168 A THR 169 A GLY 170 A ASP 187 A ARG 188 A GLN 189 A THR 190 A ALA 191 A GLN 192	−8.72
50335-04-1	A HIS 41 A MET 49 A PHE 140 A LEU 141 A ASN 142 A SER 144 A CYS 145 A HIS 163 A HIS 164 A MET 165 A GLU 166 A HIS 172 A VAL 186 A ASP 187 A ARG 188 A GLN 189 A THR 190 A GLN 192	−8.71
445430-65-9	A THR 24 A THR 25 A THR 26 A LEU 27 A HIS 41 A MET 49 A TYR 118 A ASN 119 A PHE 140 A LEU 141 A ASN 142 A GLY 143 A SER 144 A CYS 145 A HIS 163 A HIS 164 A MET 165 A GLU 166 A LEU 167 A PRO 168 A ASP 187 A ARG 188 A GLN 189 A THR 190 A GLN 192	−8.68
935841-71-7	A THR 25 A HIS 41 A MET 49 A PHE 140 A LEU 141 A ASN 142 A GLY 143 A SER 144 A CYS 145 A HIS 163 A HIS 164 A MET 165 A GLU 166 A LEU 167 A PRO 168 A HIS 172 A ASP 187 A ARG 188 A GLN 189 A THR 190 A ALA 191 A GLN 192	−8.66
136024-80-1	A THR 24 A THR 25 A THR 26 A HIS 41 A MET 49 A PHE 140 A LEU 141 A ASN 142 A GLY 143 A SER 144 A CYS 145 A HIS 163 A HIS 164 A MET 165 A GLU 166 A HIS 172 A ARG 188 A GLN 189	−8.62
852872-93-6	A THR 25 A THR 26 A LEU 27 A HIS 41 A VAL 42 A MET 49 A PHE 140 A LEU 141 A ASN 142 A GLY 143 A SER 144 A CYS 145 A HIS 163 A HIS 164 A MET 165 A GLU 166 A LEU 167 A PRO 168 A GLY 170 A ASP 187 A ARG 188 A GLN 189 A THR 190 A ALA 191 A GLN 192	−8.61
104758-18-1	A SER 46 A GLU 47 A MET 49 A LEU 50 A PHE 140 A LEU 141 A ASN 142 A SER 144 A CYS 145 A HIS 163 A HIS 164 A MET 165 A GLU 166 A LEU 167 A PRO 168 A HIS 172 A ARG 188 A GLN 189 A THR 190 A ALA 191 A GLN 192	−8.58
97915-31-6	A THR 24 A THR 25 A THR 26 A LEU 27 A HIS 41 A THR 45 A MET 49 A PHE 140 A LEU 141 A ASN 142 A GLY 143 A SER 144 A CYS 145 A HIS 163 A HIS 164 A MET 165 A GLU 166 A LEU 167 A PRO 168 A GLY 170 A HIS 172 A VAL 186 A ARG 188 A GLN 189 A THR 190 A ALA	−8.58
35906-51-5	A SER 46 A GLU 47 A MET 49 A LEU 50 A PHE 140 A LEU 141 A ASN 142 A SER 144 A CYS 145 A HIS 163 A HIS 164 A MET 165 A GLU 166 A LEU 167 A PRO 168 A HIS 172 A ARG 188 A GLN 189 A THR 190 A ALA 191 A GLN 192	−8.57

208927-15-5	A THR 24 A THR 25 A THR 26 A LEU 27 A HIS 41 A MET 49 A PHE 140 A LEU 141 A ASN 142 A GLY 143 A SER 144 A CYS 145 A HIS 163 A HIS 164 A MET 165 A GLU 166 A LEU 167 A PRO 168 A HIS 172 A GLN 189 A THR 190 A ALA 191 A GLN 192	−8.56
55544-35-9	A THR 25 A THR 26 A LEU 27 A HIS 41 A MET 49 A LEU 141 A ASN 142 A GLY 143 A SER 144 A CYS 145 A HIS 163 A MET 165 A GLU 166 A LEU 167 A PRO 168 A ARG 188 A GLN 189 A THR 190 A ALA 191 A GLN 192	−8.56
194234-73-6	A THR 24 A THR 25 A THR 26 A LEU 27 A HIS 41 A MET 49 A PHE 140 A LEU 141 A ASN 142 A GLY 143 A SER 144 A CYS 145 A HIS 163 A HIS 164 A MET 165 A GLU 166 A LEU 167 A HIS 172 A VAL 186 A ASP 187 A ARG 188 A GLN 189	−8.56
104759-19-5	A SER 46 A GLU 47 A MET 49 A LEU 50 A PHE 140 A LEU 141 A ASN 142 A SER 144 A CYS 145 A HIS 163 A HIS 164 A MET 165 A GLU 166 A LEU 167 A PRO 168 A HIS 172 A ARG 188 A GLN 189 A THR 190 A ALA 191 A GLN 192	−8.55
133613-77-1	A LEU 27 A HIS 41 A MET 49 A PHE 140 A LEU 141 A ASN 142 A GLY 143 A SER 144 A CYS 145 A HIS 163 A HIS 164 A MET 165 A GLU 166 A LEU 167 A PRO 168 A ARG 188 A GLN 189 A THR 190 A ALA 191 A GLN 192	−8.55
273410-51-8	A THR 24 A THR 25 A THR 26 A LEU 27 A HIS 41 A MET 49 A PHE 140 A LEU 141 A ASN 142 A GLY 143 A SER 144 A CYS 145 A HIS 163 A HIS 164 A MET 165 A GLU 166 A LEU 167 A VAL 186 A ASP 187 A ARG 188 A GLN 189	−8.54
115982-32-6	A THR 24 A THR 25 A THR 26 A HIS 41 A SER 46 A MET 49 A PHE 140 A LEU 141 A ASN 142 A GLY 143 A SER 144 A CYS 145 A HIS 163 A HIS 164 A MET 165 A GLU 166 A HIS 172 A VAL 186 A ASP 187 A ARG 188 A GLN 189	−8.50
81657-79-6	A THR 24 A THR 25 A THR 26 A LEU 27 A HIS 41 A SER 46 A MET 49 A TYR 54 A LEU 141 A ASN 142 A GLY 143 A SER 144 A CYS 145 A HIS 163 A HIS 164 A MET 165 A GLU 166 A VAL 186 A ASP 187 A ARG 188 A GLN 189 A GLN 192	−8.50
36119-03-6	A THR 25 A THR 26 A LEU 27 A HIS 41 A CYS 44 A MET 49 A PRO 52 A TYR 54 A PHE 140 A LEU 141 A ASN 142 A GLY 143 A SER 144 A CYS 145 A HIS 163 A HIS 164 A MET 165 A GLU 166 A PRO 168 A HIS 172 A ASP 187 A ARG 188 A GLN 189 A THR 190 A GLN 192	−8.47
83481-23-6	A MET 49 A PHE 140 A LEU 141 A ASN 142 A GLY 143 A SER 144 A CYS 145 A HIS 163 A HIS 164 A MET 165 A GLU 166 A LEU 167 A PRO 168 A HIS 172 A ARG 188 A GLN 189 A THR 190 A ALA 191 A GLN 192	−8.47
80680-43-9	A HIS 41 A MET 49 A TYR 54 A ASN 142 A CYS 145 A HIS 163 A HIS 164 A MET 165 A GLU 166 A LEU 167 A PRO 168 A ASP 187 A ARG 188 A GLN 189 A THR 190 A ALA 191	−8.47
18097-67-1	A THR 25 A THR 26 A LEU 27 A HIS 41 A CYS 44 A MET 49 A PRO 52 A TYR 54 A PHE 140 A LEU 141 A ASN 142 A GLY 143 A SER 144 A CYS 145 A HIS 163 A HIS 164 A MET 165 A GLU 166 A LEU 167 A PRO 168 A ASP 187 A ARG 188 A GLN 189	−8.46
138529-07-4	A THR 24 A THR 25 A THR 26 A LEU 27 A HIS 41 A CYS 44 A THR 45 A SER 46 A GLU 47 A ASP 48 A MET 49 A LEU 50 A TYR 54 A ASN 142 A GLY 143 A CYS 145 A HIS 163 A HIS 164 A MET 165 A GLU 166 A PRO 168 A ASP 187 A ARG 188 A GLN 189 A THR 190 A ALA 191 A	−8.45

61369-27-5	A THR 24 A THR 25 A THR 26 A HIS 41 A CYS 44 A THR 45 A SER 46 A MET 49 A TYR 54 A ASN 142 A GLY 143 A CYS 145 A HIS 164 A MET 165 A GLU 166 A PRO 168 A ASP 187 A ARG 188 A GLN 189 A THR 190 A ALA 191	−8.45
70022-71-8	A THR 25 A LEU 27 A HIS 41 A MET 49 A LEU 141 A ASN 142 A GLY 143 A SER 144 A CYS 145 A HIS 163 A HIS 164 A MET 165 A GLU 166 A LEU 167 A PRO 168 A ARG 188 A GLN 189 A THR 190 A ALA 191 A GLN 192	−8.45
55482-27-4	A THR 24 A THR 25 A THR 26 A HIS 41 A CYS 44 A THR 45 A SER 46 A MET 49 A PRO 52 A TYR 54 A PHE 140 A LEU 141 A ASN 142 A GLY 143 A SER 144 A CYS 145 A HIS 163 A HIS 164 A MET 165 A GLU 166 A LEU 167 A PRO 168 A ASP 187 A ARG 188 A GLN 189 A THR 190	−8.44
138529-05-2	A THR 24 A THR 25 A THR 26 A LEU 27 A HIS 41 A CYS 44 A THR 45 A SER 46 A MET 49 A ASN 142 A GLY 143 A CYS 145 A HIS 164 A MET 165 A GLU 166 A LEU 167 A PRO 168 A VAL 186 A ASP 187 A ARG 188 A GLN 189 A THR 190 A GLN 192	−8.40
690627-61-3	A THR 24 A THR 25 A THR 26 A LEU 27 A HIS 41 A MET 49 A PHE 140 A LEU 141 A ASN 142 A GLY 143 A SER 144 A CYS 145 A HIS 163 A HIS 164 A MET 165 A GLU 166 A LEU 167 A PRO 168 A HIS 172 A ARG 188 A GLN 189 A THR 190 A ALA 191 A GLN 192	−8.40
213547-25-2	A THR 24 A THR 25 A THR 26 A LEU 27 A HIS 41 A THR 45 A SER 46 A MET 49 A PHE 140 A LEU 141 A ASN 142 A GLY 143 A SER 144 A CYS 145 A HIS 163 A HIS 164 A MET 165 A GLU 166 A HIS 172 A GLN 189	−8.40
52796-14-2	A THR 25 A LEU 27 A HIS 41 A MET 49 A PHE 140 A LEU 141 A ASN 142 A GLY 143 A SER 144 A CYS 145 A HIS 163 A HIS 164 A MET 165 A GLU 166 A LEU 167 A PRO 168 A HIS 172 A ARG 188 A GLN 189 A THR 190 A ALA 191 A GLN 192	−8.38
207385-07-7	A THR 25 A LEU 27 A HIS 41 A MET 49 A PHE 140 A LEU 141 A ASN 142 A GLY 143 A SER 144 A CYS 145 A HIS 163 A HIS 164 A MET 165 A GLU 166 A LEU 167 A PRO 168 A HIS 172 A ARG 188 A GLN 189 A THR 190 A ALA 191 A GLN 192	-8.38
102506-55-8	A THR 24 A THR 25 A THR 26 A LEU 27 A HIS 41 A CYS 44 A THR 45 A SER 46 A MET 49 A LEU 141 A ASN 142 A GLY 143 A SER 144 A CYS 145 A MET 165 A GLU 166 A LEU 167 A PRO 168 A ARG 188 A GLN 189 A THR 190 A GLN 192	−8.38
188558-97-6	A THR 24 A THR 25 A THR 26 A HIS 41 A MET 49 A ASN 142 A GLY 143 A CYS 145 A HIS 164 A MET 165 A GLU 166 A LEU 167 A PRO 168 A ASP 187 A ARG 188 A GLN 189 A THR 190 A ALA 191 A GLN 192	−8.36
256649-68-0	A MET 49 A PHE 140 A LEU 141 A ASN 142 A GLY 143 A SER 144 A CYS 145 A HIS 163 A HIS 164 A MET 165 A GLU 166 A LEU 167 A PRO 168 A HIS 172 A ARG 188 A GLN 189 A THR 190 A GLN 192	−8.36
55945-74-9	A HIS 41 A MET 49 A PHE 140 A LEU 141 A ASN 142 A GLY 143 A SER 144 A CYS 145 A HIS 163 A HIS 164 A MET 165 A GLU 166 A LEU 167 A PRO 168 A GLN 189 A THR 190 A ALA 191	−8.34
65773-98-0	A THR 25 A THR 26 A LEU 27 A HIS 41 A MET 49 A PHE 140 A LEU 141 A ASN 142 A GLY 143 A SER 144 A CYS 145 A HIS 163 A HIS 164 A MET 165 A GLU 166 A LEU 167 A PRO 168 A HIS 172 A GLN 189	−8.34

137809-94-0	A THR 24 A THR 25 A THR 26 A LEU 27 A HIS 41 A CYS 44 A MET 49 A LEU 50 A TYR 54 A PHE 140 A LEU 141 A ASN 142 A GLY 143 A SER 144 A CYS 145 A HIS 164 A MET 165 A GLU 166 A LEU 167 A PRO 168 A GLY 170 A ASP 187 A ARG 188 A GLN 189 A THR 190 A GLN 1	−8.33
137844-97-4	A THR 24 A THR 25 A THR 26 A LEU 27 A THR 45 A SER 46 A MET 49 A PHE 140 A LEU 141 A ASN 142 A GLY 143 A SER 144 A CYS 145 A HIS 163 A HIS 164 A MET 165 A GLU 166 A LEU 167 A PRO 168 A HIS 172 A ARG 188 A GLN 189 A THR 190 A GLN 192	−8.32
36011-19-5	A THR 24 A THR 25 A THR 26 A LEU 27 A HIS 41 A VAL 42 A CYS 44 A THR 45 A SER 46 A MET 49 A PHE 140 A LEU 141 A ASN 142 A GLY 143 A SER 144 A CYS 145 A HIS 163 A HIS 164 A MET 165 A GLU 166 A HIS 172 A GLN 189	−8.30
54854-92-1	A THR 25 A THR 26 A LEU 27 A HIS 41 A MET 49 A PHE 140 A LEU 141 A ASN 142 A GLY 143 A SER 144 A CYS 145 A HIS 163 A HIS 164 A MET 165 A GLU 166 A LEU 167 A PRO 168 A HIS 172 A ARG 188 A GLN 189 A THR 190 A GLN 192	−8.29
656250-28-1	A THR 24 A THR 25 A THR 26 A HIS 41 A CYS 44 A THR 45 A SER 46 A MET 49 A PHE 140 A LEU 141 A ASN 142 A GLY 143 A SER 144 A CYS 145 A HIS 163 A HIS 164 A MET 165 A GLU 166 A HIS 172 A GLN 189	−8.29
798575-31-2	A THR 24 A THR 25 A THR 26 A HIS 41 A CYS 44 A THR 45 A SER 46 A MET 49 A PHE 140 A LEU 141 A ASN 142 A GLY 143 A SER 144 A CYS 145 A HIS 163 A HIS 164 A MET 165 A GLU 166 A HIS 172 A GLN 189	−8.29
128129-56-6	A THR 25 A THR 26 A LEU 27 A HIS 41 A MET 49 A ASN 142 A GLY 143 A CYS 145 A MET 165 A GLU 166 A VAL 186 A ASP 187 A ARG 188 A GLN 189 A THR 190 A GLN 192	−8.28
144587-58-6	A THR 25 A THR 26 A LEU 27 A HIS 41 A MET 49 A ASN 142 A GLY 143 A CYS 145 A MET 165 A GLU 166 A LEU 167 A PRO 168 A GLN 189 A THR 190 A ALA 191 A GLN 192	−8.27
72380-13-3	A THR 25 A THR 26 A LEU 27 A HIS 41 A VAL 42 A MET 49 A PHE 140 A LEU 141 A ASN 142 A GLY 143 A SER 144 A CYS 145 A HIS 163 A MET 165 A GLU 166 A LEU 167 A PRO 168 A ARG 188 A GLN 189 A THR 190 A GLN 192	−8.24
83903-60-0	A THR 25 A THR 26 A CYS 44 A THR 45 A SER 46 A MET 49 A PHE 140 A LEU 141 A ASN 142 A SER 144 A CYS 145 A HIS 163 A HIS 164 A MET 165 A GLU 166 A LEU 167 A PRO 168 A HIS 172 A ARG 188 A GLN 189 A THR 190 A ALA 191 A GLN 192	−8.23
50611-86-4	A HIS 41 A CYS 44 A MET 49 A PRO 52 A TYR 54 A PHE 140 A LEU 141 A ASN 142 A SER 144 A CYS 145 A HIS 163 A HIS 164 A MET 165 A GLU 166 A HIS 172 A ASP 187 A ARG 188 A GLN 189	−8.23
164176-22-1	A THR 24 A THR 25 A LEU 27 A HIS 41 A CYS 44 A THR 45 A SER 46 A MET 49 A PRO 52 A TYR 54 A ASN 142 A GLY 143 A CYS 145 A HIS 164 A MET 165 A GLU 166 A PHE 181 A ASP 187 A ARG 188 A GLN 189	−8.23
164176-24-3	A THR 24 A THR 25 A THR 26 A HIS 41 A CYS 44 A THR 45 A SER 46 A MET 49 A PRO 52 A TYR 54 A ASN 119 A ASN 142 A GLY 143 A CYS 145 A HIS 164 A MET 165 A GLU 166 A ASP 187 A ARG 188 A GLN 189	−8.21
70938-59-9	A HIS 41 A MET 49 A PHE 140 A LEU 141 A ASN 142 A GLY 143 A SER 144 A CYS 145 A HIS 163 A HIS 164 A MET 165 A GLU 166 A LEU 167 A PRO 168 A HIS 172 A ASP 187 A ARG 188 A GLN 189 A THR 190 A ALA 191 A GLN 192	−8.21

114-25-0 ǀ	THR 24 A THR 25 A THR 26 A HIS 41 A CYS 44 A THR 45 A SER 46 A MET 49 A PRO 52 A TYR 54 A PHE 140 A LEU 141 A ASN 142 A GLY 143 A SER 144 A CYS 145 A HIS 163 A HIS 164 A MET 165 A GLU 166 A LEU 167 A PRO 168 A ASP 187 A ARG 188 A GLN 189 A THR 190 A	−8.21
174630-08-1	A THR 24 A THR 25 A THR 26 A LEU 27 A HIS 41 A CYS 44 A THR 45 A SER 46 A MET 49 A LEU 141 A ASN 142 A GLY 143 A SER 144 A CYS 145 A HIS 163 A HIS 164 A MET 165 A GLU 166 A LEU 167 A PRO 168 A ARG 188 A GLN 189 A THR 190 A ALA 191 A GLN 192	−8.21
72509-61-6	A THR 25 A THR 26 A MET 49 A PHE 140 A LEU 141 A ASN 142 A GLY 143 A SER 144 A CYS 145 A HIS 163 A HIS 164 A MET 165 A GLU 166 A LEU 167 A PRO 168 A ARG 188 A GLN 189	−8.20
184348-39-8	A THR 25 A THR 26 A HIS 41 A MET 49 A TYR 118 A ASN 119 A ASN 142 A GLY 143 A CYS 145 A HIS 164 A MET 165 A GLU 166 A GLN 189	−8.19
137809-89-3	A THR 24 A THR 25 A LEU 27 A HIS 41 A CYS 44 A THR 45 A SER 46 A MET 49 A TYR 54 A PHE 140 A ASN 142 A GLY 143 A CYS 145 A HIS 163 A HIS 164 A MET 165 A GLU 166 A HIS 172 A ASP 187 A ARG 188 A GLN 189	−8.17
111036-40-9	A LEU 27 A HIS 41 A MET 49 A PHE 140 A LEU 141 A ASN 142 A GLY 143 A SER 144 A CYS 145 A HIS 163 A HIS 164 A MET 165 A GLU 166 A LEU 167 A PRO 168 A HIS 172 A ASP 187 A ARG 188 A GLN 189 A THR 190 A ALA 191 A GLN 192	−8.15
164176-23-2	A THR 24 A THR 25 A THR 26 A HIS 41 A CYS 44 A SER 46 A MET 49 A PRO 52 A TYR 54 A ASN 119 A ASN 142 A GLY 143 A SER 144 A CYS 145 A HIS 164 A MET 165 A GLU 166 A PHE 181 A ASP 187 A ARG 188 A GLN 189	−8.15
137824-59-0	A HIS 41 A CYS 44 A MET 49 A PRO 52 A TYR 54 A PHE 140 A LEU 141 A ASN 142 A GLY 143 A SER 144 A CYS 145 A HIS 163 A HIS 164 A MET 165 A GLU 166 A LEU 167 A PRO 168 A HIS 172 A ASP 187 A ARG 188 A GLN 189 A THR 190 A ALA 191 A GLN 192	−8.15
76689-96-8	A THR 24 A THR 25 A LEU 27 A HIS 41 A CYS 44 A THR 45 A SER 46 A MET 49 A PRO 52 A TYR 54 A ASN 142 A GLY 143 A CYS 145 A HIS 164 A MET 165 A GLU 166 A PHE 181 A ASP 187 A ARG 188 A GLN 189	−8.15
142925-51-7	A HIS 41 A CYS 44 A MET 49 A PRO 52 A TYR 54 A PHE 140 A LEU 141 A ASN 142 A SER 144 A CYS 145 A HIS 163 A HIS 164 A MET 165 A GLU 166 A LEU 167 A PRO 168 A THR 169 A GLY 170 A HIS 172 A ASP 187 A ARG 188 A GLN 189 A THR 190 A ALA 191 A GLN 192	−8.14
172854-77-2	A MET 49 A PHE 140 A LEU 141 A ASN 142 A GLY 143 A SER 144 A CYS 145 A HIS 163 A HIS 164 A MET 165 A GLU 166 A LEU 167 A PRO 168 A HIS 172 A ARG 188 A GLN 189 A THR 190 A GLN 192	−8.14
329050-25-1	A THR 24 A THR 25 A THR 26 A LEU 27 A HIS 41 A VAL 42 A MET 49 A TYR 54 A ASN 142 A GLY 143 A CYS 145 A HIS 163 A HIS 164 A MET 165 A GLU 166 A PHE 181 A ASP 187 A ARG 188 A GLN 189	−8.10
137809-88-2	A HIS 41 A CYS 44 A MET 49 A TYR 54 A PHE 140 A LEU 141 A ASN 142 A GLY 143 A SER 144 A CYS 145 A HIS 163 A MET 165 A GLU 166 A PRO 168 A HIS 172 A VAL 186 A ASP 187 A ARG 188 A GLN 189 A THR 190 A GLN 192	−8.09

86425-61-8	A THR 24 A THR 25 A THR 26 A LEU 27 A HIS 41 A VAL 42 A SER 46 A MET 49 A ASN 119 A PHE 140 A LEU 141 A ASN 142 A GLY 143 A SER 144 A CYS 145 A HIS 163 A HIS 164 A MET 165 A GLU 166 A HIS 172 A ASP 187 A ARG 188 A GLN 189	−8.06
823808-55-5	A THR 25 A LEU 27 A HIS 41 A MET 49 A ASN 142 A GLY 143 A SER 144 A CYS 145 A HIS 163 A HIS 164 A MET 165 A GLU 166 A LEU 167 A PRO 168 A HIS 172 A ARG 188 A GLN 189 A THR 190 A ALA 191 A GLN 192	−8.06
145613-82-7	A LEU 27 A HIS 41 A MET 49 A PHE 140 A LEU 141 A ASN 142 A GLY 143 A SER 144 A CYS 145 A HIS 163 A HIS 164 A MET 165 A GLU 166 A LEU 167 A PRO 168 A HIS 172 A ASP 187 A ARG 188 A GLN 189 A THR 190 A ALA 191 A GLN 192	−8.05
858950-52-4	A THR 25 A THR 26 A LEU 27 A HIS 41 A MET 49 A ASN 142 A GLY 143 A SER 144 A CYS 145 A HIS 163 A HIS 164 A MET 165 A GLU 166 A PRO 168 A GLN 189	−8.05
80442-78-0	A MET 49 A LEU 141 A ASN 142 A GLY 143 A SER 144 A CYS 145 A HIS 163 A HIS 164 A MET 165 A GLU 166 A LEU 167 A PRO 168 A GLN 189 A THR 190	−8.04
80442-79-1	A MET 49 A LEU 141 A ASN 142 A GLY 143 A SER 144 A CYS 145 A HIS 163 A HIS 164 A MET 165 A GLU 166 A LEU 167 A PRO 168 A GLN 189 A THR 190	−8.04
115268-43-4	A HIS 41 A MET 49 A LEU 141 A ASN 142 A CYS 145 A HIS 163 A HIS 164 A MET 165 A GLU 166 A LEU 167 A PRO 168 A ARG 188 A GLN 189	−8.04
364344-14-9	A THR 24 A THR 25 A THR 26 A LEU 27 A HIS 41 A MET 49 A LEU 141 A ASN 142 A GLY 143 A SER 144 A CYS 145 A HIS 163 A HIS 164 A MET 165 A GLU 166 A LEU 167 A PRO 168 A ARG 188 A GLN 189 A THR 190 A GLN 192	−8.04
105404-85-1	A THR 24 A THR 25 A THR 26 A HIS 41 A CYS 44 A THR 45 A SER 46 A MET 49 A LEU 141 A ASN 142 A GLY 143 A SER 144 A CYS 145 A HIS 163 A HIS 164 A MET 165 A GLU 166 A LEU 167 A PRO 168 A ARG 188 A GLN 189 A THR 190	−8.04
137824-58-9	A THR 25 A THR 26 A LEU 27 A MET 49 A PHE 140 A LEU 141 A ASN 142 A GLY 143 A SER 144 A CYS 145 A HIS 163 A HIS 164 A MET 165 A GLU 166 A PRO 168 A HIS 172 A ARG 188 A GLN 189 A THR 190 A ALA 191 A GLN 192	−8.04
99102-40-6	A THR 24 A THR 25 A THR 26 A LEU 27 A HIS 41 A VAL 42 A THR 45 A SER 46 A MET 49 A TYR 118 A ASN 142 A GLY 143 A CYS 145 A MET 165 A GLU 166 A GLN 189	−8.02
100244-36-8	A THR 24 A THR 25 A THR 26 A LEU 27 A HIS 41 A MET 49 A LEU 141 A ASN 142 A GLY 143 A SER 144 A CYS 145 A HIS 163 A HIS 164 A MET 165 A GLU 166 A GLN 189	−8.02
157207-91-5	A MET 49 A PHE 140 A LEU 141 A ASN 142 A GLY 143 A SER 144 A CYS 145 A HIS 163 A HIS 164 A MET 165 A GLU 166 A LEU 167 A PRO 168 A HIS 172 A ASP 187 A ARG 188 A GLN 189 A THR 190 A ALA 191	−8.02
329197-89-9	A THR 24 A THR 25 A THR 26 A LEU 27 A HIS 41 A MET 49 A LEU 141 A ASN 142 A GLY 143 A SER 144 A CYS 145 A HIS 163 A HIS 164 A MET 165 A GLU 166 A GLN 189	−8.01
2643-02-9 |	THR 24 A THR 25 A THR 26 A LEU 27 A HIS 41 A THR 45 A SER 46 A MET 49 A LEU 141 A ASN 142 A GLY 143 A SER 144 A CYS 145 A HIS 163 A HIS 164 A MET 165 A GLU 166 A ARG 188 A GLN 189 A GLN 192	-8.01
143346-97-8	A HIS 41 A MET 49 A PHE 140 A LEU 141 A ASN 142 A GLY 143 A SER 144 A CYS 145 A HIS 164 A MET 165 A GLU 166 A LEU 167 A PRO 168 A ARG 188 A GLN 189 A THR 190 A ALA 191 A GLN 192	−8.01

149764-33-0	A HIS 41 A MET 49 A LEU 50 A PHE 140 A LEU 141 A ASN 142 A GLY 143 A SER 144 A CYS 145 A HIS 163 A HIS 164 A MET 165 A GLU 166 A LEU 167 A PRO 168 A HIS 172 A GLN 189 A THR 190 A ALA 191 A GLN 192	−8.00
143113-32-0	A THR 24 A THR 25 A THR 26 A LEU 27 A HIS 41 A THR 45 A SER 46 A MET 49 A PHE 140 A LEU 141 A ASN 142 A GLY 143 A SER 144 A CYS 145 A HIS 163 A HIS 164 A MET 165 A GLU 166 A LEU 167 A PRO 168 A GLY 170 A HIS 172 A GLN 189	−7.99
77658-95-8	A THR 24 A THR 25 A THR 26 A LEU 27 A HIS 41 A CYS 44 A MET 49 A TYR 54 A PHE 140 A LEU 141 A ASN 142 A GLY 143 A SER 144 A CYS 145 A HIS 163 A HIS 164 A MET 165 A GLU 166 A HIS 172 A ASP 187 A ARG 188 A GLN 189	−7.99
858950-36-4	A PHE 140 A LEU 141 A ASN 142 A GLY 143 A SER 144 A CYS 145 A HIS 163 A HIS 164 A MET 165 A GLU 166 A PRO 168 A HIS 172 A GLN 189 A THR 190 A ALA 191 A GLN 192	−7.98
79206-71-6	A THR 25 A HIS 41 A MET 49 A PHE 140 A LEU 141 A ASN 142 A GLY 143 A SER 144 A CYS 145 A HIS 163 A HIS 164 A MET 165 A GLU 166 A LEU 167 A PRO 168 A HIS 172 A ARG 188 A GLN 189 A THR 190 A ALA 191 A GLN 192	−7.98
724462-47-9	A THR 25 A THR 26 A LEU 27 A HIS 41 A MET 49 A TYR 54 A PHE 140 A LEU 141 A ASN 142 A GLY 143 A SER 144 A CYS 145 A HIS 163 A HIS 164 A MET 165 A GLU 166 A HIS 172 A ASP 187 A ARG 188 A GLN 189	−7.97
2122-98-7 |	THR 25 A THR 26 A LEU 27 A HIS 41 A MET 49 A LEU 141 A ASN 142 A GLY 143 A SER 144 A CYS 145 A HIS 163 A HIS 164 A MET 165 A GLU 166 A HIS 172 A GLN 189 A THR 190	−7.96
79067-75-7	A THR 24 A THR 25 A THR 26 A LEU 27 A SER 46 A MET 49 A ASN 119 A PHE 140 A LEU 141 A ASN 142 A GLY 143 A SER 144 A CYS 145 A HIS 163 A GLU 166 A GLN 189	−7.92
603125-81-1	A THR 25 A THR 26 A LEU 27 A HIS 41 A MET 49 A ASN 142 A GLY 143 A SER 144 A CYS 145 A HIS 164 A MET 165 A GLU 166 A PRO 168 A ARG 188 A GLN 189 A THR 190 A GLN 192	−7.90
134985-04-9	A HIS 41 A CYS 44 A MET 49 A LEU 50 A PRO 52 A TYR 54 A PHE 140 A LEU 141 A ASN 142 A GLY 143 A SER 144 A CYS 145 A HIS 163 A HIS 164 A MET 165 A GLU 166 A LEU 167 A PRO 168 A HIS 172 A ASP 187 A ARG 188 A GLN 189 A THR 190 A ALA 191	−7.90
94513-77-6	A THR 24 A THR 25 A THR 26 A LEU 27 A HIS 41 A CYS 44 A THR 45 A SER 46 A MET 49 A LEU 50 A TYR 54 A CYS 145 A HIS 164 A MET 165 A GLU 166 A LEU 167 A ASP 187 A ARG 188 A GLN 189 A THR 190 A ALA 191 A GLN 192	−7.90
92679-03-3	A THR 24 A THR 25 A THR 26 A LEU 27 A HIS 41 A MET 49 A TYR 54 A ASN 142 A GLY 143 A CYS 145 A HIS 164 A MET 165 A GLU 166 A ASP 187 A ARG 188 A GLN 189	−7.89
65604-87-7	A PHE 140 A LEU 141 A ASN 142 A GLY 143 A SER 144 A CYS 145 A HIS 163 A HIS 164 A MET 165 A GLU 166 A LEU 167 A PRO 168 A GLY 170 A HIS 172 A ASP 187 A ARG 188 A GLN 189 A THR 190 A GLN 192	−7.89
304902-16-7	A HIS 41 A MET 49 A PHE 140 A LEU 141 A ASN 142 A GLY 143 A SER 144 A CYS 145 A HIS 163 A HIS 164 A MET 165 A GLU 166 A LEU 167 A PRO 168 A ARG 188 A GLN 189 A THR 190 A ALA 191 A GLN 192	−7.88

181034-78-6	A HIS 41 A CYS 44 A MET 49 A PRO 52 A TYR 54 A ASN 142 A CYS 145 A HIS 164 A MET 165 A GLU 166 A LEU 167 A PRO 168 A GLY 170 A ASP 187 A ARG 188 A GLN 189 A THR 190 A ALA 191 A GLN 192	−7.87
221290-77-3	A THR 24 A THR 25 A THR 26 A LEU 27 A HIS 41 A MET 49 A TYR 54 A ASN 119 A ASN 142 A GLY 143 A CYS 145 A HIS 164 A MET 165 A GLU 166 A LEU 167 A PRO 168 A ASP 187 A ARG 188 A GLN 189 A THR 190 A ALA 191 A GLN 192	−7.87
76689-97-9	A HIS 41 A MET 49 A PHE 140 A LEU 141 A ASN 142 A GLY 143 A SER 144 A CYS 145 A HIS 163 A HIS 164 A MET 165 A GLU 166 A LEU 167 A PRO 168 A HIS 172 A ASP 187 A ARG 188 A GLN 189 A THR 190 A ALA 191	−7.86
92679-02-2	A THR 24 A THR 25 A THR 26 A LEU 27 A HIS 41 A CYS 44 A MET 49 A PRO 52 A TYR 54 A ASN 142 A GLY 143 A CYS 145 A HIS 163 A HIS 164 A MET 165 A GLU 166 A PHE 181 A ASP 187 A ARG 188 A GLN 189	−7.86
170894-36-7	A THR 25 A THR 26 A LEU 27 A HIS 41 A MET 49 A LEU 50 A ASN 142 A GLY 143 A CYS 145 A HIS 164 A MET 165 A GLU 166 A LEU 167 A PRO 168 A GLN 189 A THR 190 A ALA 191 A GLN 192	−7.85
170894-37-8	A THR 25 A THR 26 A LEU 27 A HIS 41 A MET 49 A ASN 142 A GLY 143 A CYS 145 A HIS 164 A MET 165 A GLU 166 A LEU 167 A PRO 168 A GLN 189 A THR 190 A ALA 191 A GLN 192	−7.84
564-16-9 ǀ	MET 49 A PHE 140 A LEU 141 A ASN 142 A GLY 143 A SER 144 A CYS 145 A HIS 163 A HIS 164 A MET 165 A GLU 166 A LEU 167 A PRO 168 A HIS 172 A GLN 189 A THR 190 A ALA 191 A GLN 192	−7.84
129602-18-2	A THR 25 A THR 26 A LEU 27 A HIS 41 A VAL 42 A MET 49 A PHE 140 A LEU 141 A ASN 142 A GLY 143 A CYS 145 A HIS 163 A HIS 164 A MET 165 A GLU 166 A LEU 167 A PRO 168 A HIS 172 A GLN 189 A THR 190 A ALA 191 A GLN 192	−7.84
142797-28-2	A PHE 140 A LEU 141 A ASN 142 A GLY 143 A SER 144 A CYS 145 A HIS 163 A HIS 164 A MET 165 A GLU 166 A LEU 167 A PRO 168 A HIS 172 A ARG 188 A GLN 189 A THR 190 A GLN 192	−7.83
58115-30-3	A THR 25 A THR 26 A LEU 27 A HIS 41 A MET 49 A PHE 140 A LEU 141 A ASN 142 A GLY 143 A SER 144 A CYS 145 A HIS 163 A MET 165 A GLU 166 A LEU 167 A PRO 168 A ARG 188 A GLN 189 A THR 190 A ALA 191 A GLN 192	−7.83
53755-56-9	A THR 25 A THR 26 A LEU 27 A HIS 41 A MET 49 A PHE 140 A LEU 141 A ASN 142 A GLY 143 A SER 144 A CYS 145 A HIS 163 A HIS 164 A MET 165 A GLU 166 A LEU 167 A PRO 168 A HIS 172 A VAL 186 A ASP 187 A ARG 188 A GLN 189 A THR 190 A ALA 191 A GLN 192	−7.83
123167-01-1	A THR 24 A THR 25 A THR 26 A LEU 27 A HIS 41 A THR 45 A SER 46 A MET 49 A LEU 141 A ASN 142 A GLY 143 A SER 144 A CYS 145 A HIS 163 A HIS 164 A MET 165 A GLU 166 A LEU 167 A PRO 168 A ARG 188 A GLN 189 A THR 190 A ALA 191 A GLN 192	−7.83
603125-80-0	A THR 25 A THR 26 A LEU 27 A HIS 41 A MET 49 A ASN 142 A GLY 143 A SER 144 A CYS 145 A HIS 164 A MET 165 A GLU 166 A LEU 167 A PRO 168 A ARG 188 A GLN 189 A THR 190 A ALA 191 A GLN 192	−7.83

207683-70-3	A THR 25 A THR 26 A LEU 27 A HIS 41 A SER 46 A MET 49 A LEU 141 A ASN 142 A GLY 143 A SER 144 A CYS 145 A HIS 163 A MET 165 A GLU 166 A LEU 167 A PRO 168 A ARG 188 A GLN 189 A THR 190 A GLN 192	−7.83
129893-35-2	A PHE 140 A LEU 141 A ASN 142 A GLY 143 A SER 144 A CYS 145 A HIS 163 A HIS 164 A MET 165 A GLU 166 A LEU 167 A PRO 168 A HIS 172 A ARG 188 A GLN 189 A THR 190 A GLN 192	−7.83
137571-30-3	A THR 24 A THR 25 A THR 26 A LEU 27 A HIS 41 A SER 46 A MET 49 A LEU 141 A ASN 142 A GLY 143 A SER 144 A CYS 145 A HIS 163 A HIS 164 A MET 165 A GLU 166 A ARG 188 A GLN 189 A THR 190	−7.82
79005-82-6	A THR 24 A THR 25 A THR 26 A LEU 27 A HIS 41 A CYS 44 A THR 45 A SER 46 A MET 49 A PHE 140 A LEU 141 A ASN 142 A GLY 143 A SER 144 A CYS 145 A HIS 163 A HIS 164 A MET 165 A GLU 166 A HIS 172 A GLN 189	−7.82
128298-63-5	A PHE 140 A LEU 141 A ASN 142 A GLY 143 A SER 144 A CYS 145 A HIS 163 A HIS 164 A MET 165 A GLU 166 A LEU 167 A PRO 168 A HIS 172 A ARG 188 A GLN 189 A THR 190 A GLN 192	−7.82
64108-85-6	A THR 24 A THR 25 A THR 26 A LEU 27 A HIS 41 A CYS 44 A SER 46 A MET 49 A PRO 52 A TYR 54 A ASN 142 A GLY 143 A CYS 145 A HIS 164 A MET 165 A GLU 166 A ASP 187 A ARG 188 A GLN 189	−7.82
123154-33-6	A THR 25 A LEU 27 A HIS 41 A MET 49 A PHE 140 A LEU 141 A ASN 142 A GLY 143 A SER 144 A CYS 145 A HIS 163 A HIS 164 A MET 165 A GLU 166 A LEU 167 A PRO 168 A GLY 170 A ASP 187 A ARG 188 A GLN 189 A THR 190 A ALA 191 A GLN 192	−7.82
100019-24-7	A PHE 140 A LEU 141 A ASN 142 A GLY 143 A SER 144 A CYS 145 A HIS 163 A HIS 164 A MET 165 A GLU 166 A LEU 167 A PRO 168 A HIS 172 A ARG 188 A GLN 189 A THR 190 A GLN 192	−7.81
128733-04-0	A MET 49 A PHE 140 A LEU 141 A ASN 142 A SER 144 A CYS 145 A HIS 163 A HIS 164 A MET 165 A GLU 166 A PRO 168 A HIS 172 A ARG 188 A GLN 189 A THR 190 A ALA 191 A GLN 192	−7.79
137809-87-1	A THR 24 A THR 25 A THR 26 A HIS 41 A CYS 44 A THR 45 A SER 46 A MET 49 A PHE 140 A LEU 141 A ASN 142 A GLY 143 A SER 144 A CYS 145 A HIS 163 A HIS 164 A MET 165 A GLU 166 A HIS 172	−7.78
128819-90-9	A MET 49 A PHE 140 A LEU 141 A ASN 142 A SER 144 A CYS 145 A HIS 163 A HIS 164 A MET 165 A GLU 166 A HIS 172 A ARG 188 A GLN 189 A THR 190 A ALA 191 A GLN 192	−7.78
80764-19-8	A THR 25 A HIS 41 A MET 49 A TYR 54 A PHE 140 A LEU 141 A ASN 142 A GLY 143 A SER 144 A CYS 145 A HIS 163 A HIS 164 A MET 165 A GLU 166 A LEU 167 A PRO 168 A HIS 172 A ASP 187 A ARG 188 A GLN 189 A THR 190 A ALA 191 A GLN 192	−7.78
160796-24-7	A THR 25 A THR 26 A LEU 27 A HIS 41 A CYS 44 A THR 45 A SER 46 A MET 49 A PHE 140 A LEU 141 A ASN 142 A GLY 143 A SER 144 A CYS 145 A HIS 163 A HIS 164 A MET 165 A GLU 166 A HIS 172 A GLN 189	−7.77
133056-09-4	A THR 24 A THR 25 A THR 26 A LEU 27 A HIS 41 A THR 45 A SER 46 A MET 49 A ASN 142 A GLY 143 A CYS 145 A MET 165 A GLU 166 A LEU 167 A PRO 168 A GLN 189	−7.77

23235-67-8	A HIS 41 A MET 49 A PHE 140 A LEU 141 A ASN 142 A GLY 143 A SER 144 A CYS 145 A HIS 163 A HIS 164 A MET 165 A GLU 166 A LEU 167 A PRO 168 A HIS 172 A ASP 187 A ARG 188 A GLN 189 A THR 190 A GLN 192	−7.76
681260-09-3	A HIS 41 A MET 49 A ASN 142 A GLY 143 A CYS 145 A HIS 163 A HIS 164 A MET 165 A GLU 166 A PRO 168 A ARG 188 A GLN 189 A THR 190 A ALA 191 A GLN 192	−7.76
33579-63-4	A THR 25 A THR 26 A LEU 27 A HIS 41 A VAL 42 A MET 49 A PHE 140 A LEU 141 A ASN 142 A GLY 143 A SER 144 A CYS 145 A HIS 163 A HIS 164 A MET 165 A GLU 166 A HIS 172 A ARG 188 A GLN 189	−7.76
648883-31-2	A THR 25 A THR 26 A LEU 27 A HIS 41 A MET 49 A LEU 141 A ASN 142 A GLY 143 A SER 144 A CYS 145 A HIS 164 A MET 165 A GLU 166 A PRO 168 A ARG 188 A GLN 189 A THR 190 A GLN 192	−7.75
143572-73-0	A THR 24 A THR 25 A THR 26 A LEU 27 A HIS 41 A MET 49 A TYR 54 A PHE 140 A LEU 141 A ASN 142 A GLY 143 A CYS 145 A HIS 163 A HIS 164 A MET 165 A GLU 166 A PHE 181 A ASP 187 A ARG 188 A GLN 189	−7.75
159334-35-7	A HIS 41 A MET 49 A PHE 140 A LEU 141 A ASN 142 A GLY 143 A SER 144 A CYS 145 A HIS 163 A HIS 164 A MET 165 A GLU 166 A PRO 168 A HIS 172 A GLN 189 A THR 190 A ALA 191 A GLN 192	−7.75
184885-91-4	A THR 25 A THR 26 A LEU 27 A HIS 41 A CYS 44 A THR 45 A SER 46 A MET 49 A PHE 140 A LEU 141 A ASN 142 A GLY 143 A SER 144 A CYS 145 A HIS 163 A HIS 164 A MET 165 A GLU 166 A HIS 172 A GLN 189	−7.74
133883-22-4	A THR 25 A THR 26 A LEU 27 A MET 49 A PHE 140 A LEU 141 A ASN 142 A GLY 143 A SER 144 A CYS 145 A HIS 163 A HIS 164 A MET 165 A GLU 166 A LEU 167 A PRO 168 A HIS 172 A ARG 188 A GLN 189 A THR 190 A ALA 191 A GLN 192	−7.74
